# Fluid shear driven secondary nucleation using α-and γ-glycine seeds confirmed with rigorous control experiments

**DOI:** 10.1039/d6ce00279j

**Published:** 2026-04-22

**Authors:** Lucas Nahas, Mei Lee, Mark D. Haw, Jan Sefcik

**Affiliations:** a Department of Chemical and Process Engineering, University of Strathclyde Glasgow G1 1XJ UK lucas.nahas-martinez.2017@uni.strath.ac.uk jan.sefcik@strath.ac.uk; b Substance Development – Materials Science Medicine Development & Supply, R&D, GSK Stevenage SG1 2NY UK; c CMAC, University of Strathclyde Glasgow G1 1RD UK

## Abstract

The effect of fluid shear on secondary nucleation has long been debated in the crystallization literature. In this work, we investigated the influence of seed polymorphism on secondary nucleation under flow conditions in the absence of mechanical impact, using a “seed-on-a-stick” technique to isolate the effect of fluid shear on secondary nucleation induced by a single glycine seed crystal in aqueous solutions. *In situ* imaging and particle count analysis were used to assess the impact of seed polymorphism on secondary nucleation kinetics. The presence of glycine seeds induced earlier nucleation under all conditions investigated here, as evidenced by shorter delay times compared to those for control and unseeded experiments. By seeding with either α- or γ-glycine, we found that the solid form of the seed had no significant impact on secondary nucleation rates or delay times and α-glycine consistently nucleated irrespective of the seed solid form. This indicates that cross-nucleation of metastable α-glycine can occur with γ-glycine seeds under flow conditions. We propose that fluid shear driven secondary nucleation may operate similarly to primary nucleation near a solution–solid interface, where enhancement of nucleation is due to stabilisation and aggregation of solute clusters in the interfacial solution region.

## Abstract 2

We propose that fluid shear driven secondary nucleation may operate similarly to primary nucleation near solution-solid interface, where enhancement of nucleation is due to stabilisation and aggregation of solute clusters in its vicinity.

## Graphical abstract

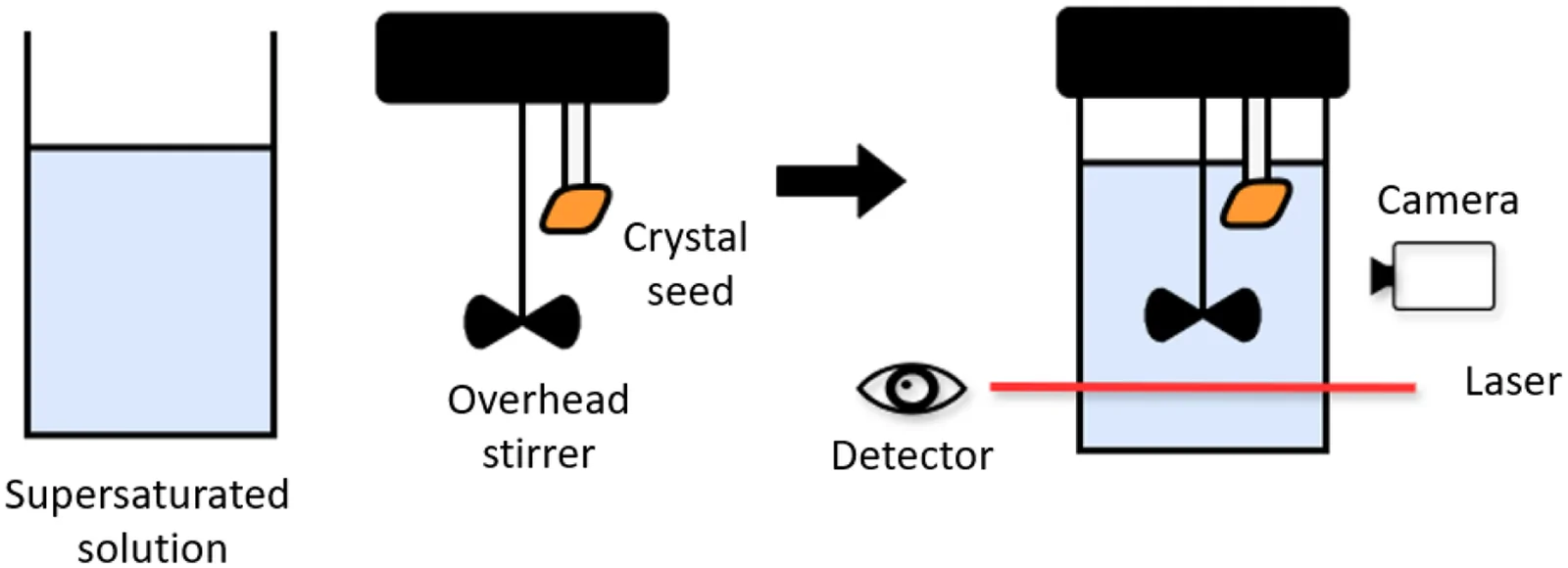

## 1 Introduction

Solid form is an important attribute to control in the production of active pharmaceutical ingredients (APIs) as different polymorphs exhibit distinct physicochemical properties such as solubility, density or crystal morphology. Undesired polymorphs can complicate downstream processes, like filtration, drying or compaction, and decrease bioavailability, reducing the efficacy of the drug product and posing health safety concerns for the patient.^1^ Therefore, to ensure consistent, stable and effective pharmaceutical products, rigorous polymorphic control is necessary.

To control polymorphism, seed crystals with a defined particle size distribution, mass loading ratio and solid form composition are usually added into a solution in its metastable zone to either induce growth of the introduced seeds or promote earlier nucleation of new crystals.^2^ Nevertheless, despite being widely used, seeding techniques can sometimes fail to target the desired solid form,^3^ as the crystallization outcome depends not only on the polymorphic composition of the seeds, but also on the relative rates of nucleation and growth of the various solid forms.^4^

During nucleation, as new crystals are formed from solution, there is a chance of different solid forms being generated concomitantly. However, despite their critical role in the polymorphic outcome, nucleation processes remain poorly understood. Nucleation mechanisms can be classified into primary or secondary types. Primary nucleation refers to the spontaneous creation of crystal nuclei directly from solution (homogeneous) or at interfaces such as container walls or stirrers (heterogeneous), whereas secondary nucleation involves the formation of new crystals due to the presence of pre-existing crystalline solids of the same substance.

Secondary nucleation is usually the dominant nucleation mechanism in seeded crystallization. Nevertheless, most research on polymorphism focuses on the influence of solvent,^5,6^ impurities,^7,8^ crystallization methods^9,10^ and operating conditions^11,12^ in unseeded crystallization, neglecting the role of seed crystals. For instance, Liu *et al.*^13^ demonstrated that higher supersaturation levels promote the formation of the metastable form of *m*-hydroxybenzoic acid (form II) in cooling crystallization from propanol, independent of the agitation rate, which is consistent with Ostwald's rule of stages. In the case of l-glutamic acid, lower temperatures favoured its metastable form in aqueous solutions,^14^ while the effect of agitation yielded contradictory results.^15,16^ On the other hand, for glycine, agitation favoured the formation of its metastable form (α) in aqueous solutions regardless of supersaturation.^17^ This variability illustrates that polymorphism is a system-specific phenomenon and factors like supersaturation, temperature or agitation can affect different systems uniquely even if the same solvent is employed.

Therefore, the influence of seeds should also be considered as a variable that can affect polymorphic outcome. Seeding with a target solid form does not necessarily ensure crystallization of the same polymorph, as the introduced seeds can also promote the formation of a different phase through a mechanism known as secondary cross-nucleation.

Different types of cross-nucleation phenomena have been previously reported in the literature. The most common involves the nucleation and growth of a faster-growing polymorph on the surface of a slower-growing seed.^18^ This behaviour has been predominantly observed in stagnant melt crystallization and is well-documented for polymers like isotactic polybutene (i-PBU), where form II crystals were reported to nucleate on the surface of form I spherulites.^19^ Nevertheless, such mechanisms have also been observed in small-molecule systems, with notable examples including d-mannitol,^20^l-glutamic acid,^21^ and ROY.^22^

In all of these cases, crystals of a different solid form were observed to grow on the surface of seeds of the same compound under stagnant conditions. Notably, this cross-nucleation behaviour is not governed by polymorph stability and does not necessarily conform to Ostwald's rule of stages. Instead, it appears to be dictated by differences in growth kinetics, where a faster-growing polymorph can overtake a slower-growing one, regardless of their relative thermodynamic stabilities, as discussed by Yu *et al.*^18^

Alternatively, cross-nucleation can also be induced by mechanical contact, as demonstrated by Cui *et al.*,^23^ where a single γ-glycine crystal was subjected to a controlled impact within a supersaturated solution. In that study, crystals of the same solid form as the seed (γ) were only generated at high impact forces (>2 N), while crystals of the metastable form (α) were formed under both high and low impact conditions, suggesting that γ-glycine seeds can induce secondary-cross nucleation of α-glycine crystals through the effect of mechanical impact.

Lastly, cross-nucleation under flow conditions, in the absence of mechanical impact, has also been reported before for chiral crystals like sodium chlorate.^24–26^ In these studies, the influence of fluid shear was isolated from mechanical impact using a seed-on-a-stick approach, where a single d-enantiomer NaClO_3_ crystal was fixed within an agitated supersaturated solution. Despite the use of d-enantiomer seeds, l-enantiomer crystals were observed to nucleate, suggesting that secondary nuclei originated from the solution layer adjacent to the seed (the boundary layer) rather than the parent crystal and were subsequently displaced by fluid flow. These findings motivated the development of new secondary nucleation models that incorporate mechanisms beyond simple attrition, commonly referred to as surface or nuclei breeding.

In this context, two main theoretical frameworks were proposed to explain nucleation induced at the interface of a seed crystal: embryo coagulation secondary nucleation (ECSN) and secondary nucleation by interparticle energies (SNIPE). The ECSN model^27^ suggests that solute clusters in solution are attracted to the seed surface through long range van der Waals forces, leading to an accumulation of clusters in the vicinity of the seed, increasing the local solute concentration and promoting a rapid coagulation that produces secondary nuclei larger than the critical size. This model, however, simplifies interparticle interactions, as it only considers van der Waals forces, neglecting the effect of electrostatic interactions for ions.

To address these limitations, the recently developed SNIPE model^28–30^ provides a more general description of the interactions that can stabilize solute clusters near the seed surface. According to this theory, such interactions reduce the Gibbs free energy required for cluster formation within a defined interfacial region, decreasing the nucleation energy barrier. Consequently, secondary nucleation is treated as an enhanced form of primary nucleation driven by local cluster stabilization rather than by coagulation.

Regardless of the exact mechanism that dominates secondary nucleation, both models could provide a plausible explanation for the formation of secondary nuclei with different chiral or polymorphic forms, as the stabilization or coagulation processes that are hypothesized to occur in the boundary layer are not necessarily dependent on the parent crystal structure. Instead, they depend on the formation or coagulation of solute clusters in the solution layer adjacent to the seed crystal.

Therefore, given the influence that secondary nucleation mechanisms can have on the polymorphic outcome, a more fundamental understanding of secondary nucleation is essential to mitigate the formation of undesired solid forms in seeded crystallization. Seeded crystallization can be dominated by growth or secondary nucleation, depending on factors such as seed loading ratio, size distribution or supersaturation.^31^ If seeded crystallization is dominated by secondary nucleation, there is a higher chance of generating nuclei of a different solid form.^20^ To avoid this, previous studies focused primarily on both model-based and model-free strategies that promote the controlled growth of seeds instead in order to target the desired solid form.^32,33^ Nevertheless, industrial constraints on yield or seed loading ratios can often lead to unavoidable secondary nucleation events, even at low supersaturation levels.^34^

In the literature, two general ideas have been proposed to explain secondary nucleation, based on the origin of secondary nuclei. According to these theories, secondary nuclei can arise from either the detachment of fragments from parent crystals by mechanical impact (attrition) or from the displacement of nucleation precursors from growing crystal surfaces by fluid shear or mechanical contact.^35^

It is argued that secondary nuclei originating from mechanical fragmentation maintain the parent crystal's solid form, unless solvent-mediated transformation occurs, whereas solute clusters displaced from the solution layer adjacent to a growing seed (boundary layer) can lead to a different solid form.^36^ Therefore, developing experimental methods capable of distinguishing these effects is essential to better understand the relationship between secondary nucleation and polymorphism. However, decoupling mechanical impact from fluid shear effects remains experimentally challenging, as it has been suggested that crystal collision forces can lead to both attrition of the parent seed and solute cluster displacement at the same time.^37^

In this study, we adopted a “seed-on-a-stick” technique to investigate the effect of fluid shear in the absence of mechanical impact. Similar techniques were used in some previous studies,^24,26,38,39^ and a need for diligently executed control experiments was recently highlighted to avoid overestimating the capability of fluid shear in inducing secondary nucleation.^40^ With this technique, the motion of the seed is restricted by fixing a single crystal within an agitated, supersaturated solution. Therefore, by isolating the influence of fluid dynamics we were able to test if fluid-shear alone can drive secondary nucleation and if it can be responsible for a change in polymorphism. Seeding with either γ- or α-glycine seeds allowed us to explore how seed polymorphism can affect both secondary nucleation kinetics and polymorphic outcome. Hence, our experimental approach addressed two key aspects: (1) the impact that fluid shear can have on secondary nucleation kinetics confirmed by rigorous control experiments and (2) the influence that fluid shear can have on polymorphism in secondary nucleation and, specifically, its potential for cross-nucleation with α- and γ-glycine seed crystals.

## 2 Methodology

In this work, small scale (6 mL) batch isothermal crystallization was conducted in aqueous glycine solutions using single-seed, control and unseeded approaches under the conditions outlined in Table 1. The glycine–water system was selected due to its well-characterized solubility^41^ and growth kinetics.^42^ Glycine has two main accessible solid forms under ambient conditions: the stable γ-form and the metastable α-form. Previous studies have shown that α-glycine undergoes solvent-mediated transformation to the γ-form after a minimum period of 10 hours.^43^ Therefore, polymorphic changes resulting from this effect were ruled out in all experiments described herein.

**Table 1:** Crystallization conditions at different temperatures and concentrations

Temperature (°C)	25	20
Concentration (mg g^−1^)	295–310–320	320
Seeded with γ	✓	✓
Seeded with α	✓	✗
Control	✓	✓
Unseeded	✓	✓

### 2.1 Materials and instruments

Glycine, supplied by Sigma-Aldrich (≥99% electrophoresis), and deionized water, sourced on-site (Milli-Q, 18.2 MΩ cm), were used. Commercial glycine was re-crystallized to obtain seeds of the α and γ forms and subsequently characterized using a Raman XplorA microscope from Horiba Scientific, equipped with an Ondax Thz-Raman probe module. Control, unseeded, and seeded crystallization experiments were performed in a Crystalline platform provided by Technobis and equipped with *in situ* visual monitoring. The in-built imaging and image analysis algorithm of the Crystalline instrument were used to obtain crystal count and detection time data. Products from seeded crystallization were characterized using a Bruker Tensor II ATR-IR spectrometer.

### 2.2 Seed preparation and characterization

A solution containing 400 mg of glycine per g of water was prepared to obtain α-glycine seeds. The mixture was dissolved at 60 °C and 400 rpm for 1 hour and subsequently cooled to 20 °C at a cooling rate of −5 °C min^−1^, at which point stirring ceased and crystals were allowed to form on the surface of the PTFE bar. Alternatively, γ-glycine seeds were formed from a brine solution (12 g NaCl per 100 g water) saturated with 30 g of glycine. The solution was left in an incubator at 25 °C for 3 days, leading to the formation of γ-glycine seeds *via* evaporative crystallization.^44^ All seeds were characterized using Raman spectroscopy, which measures inelastic scattering from a 532 nm laser focused on the crystal surface. The terahertz region (50–200 cm^−1^) of the Raman spectrum was analysed to differentiate solid forms, with γ-glycine identified by characteristic peaks at 91 and 154 cm^−1^.^45^

### 2.3 Seed-on-a-stick (SOAS) experiments

**Fig. 1:**
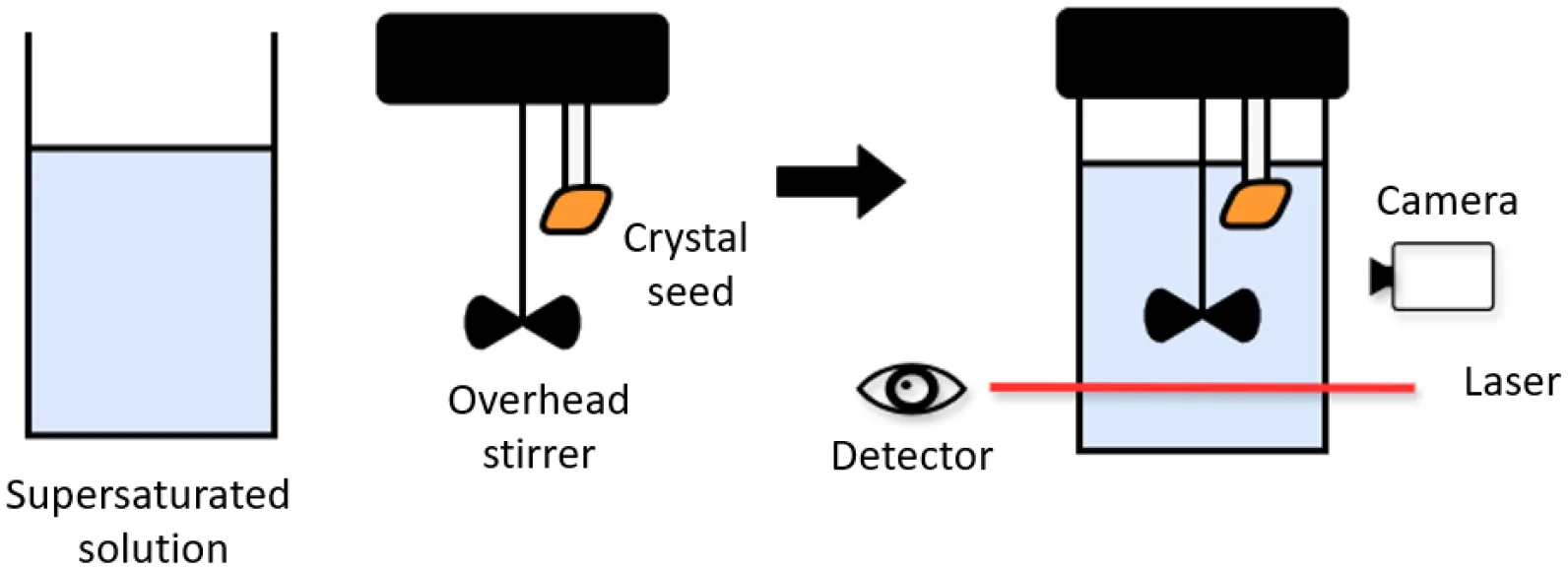
Seed-on-a-stick set-up in a Crystalline cylindrical vial. A laser and detector were used to detect the onset of nucleation by transmissivity measurements. The camera was used with the in-built image analysis algorithm to count the number of crystals in each image, used to measure secondary nucleation rate.

Seed crystals of the desired solid form with a size of ∼2 mm were glued to capillary tubes and positioned at a distance of 4 mm from the impeller tip as shown in Fig. 1. To prevent initial breeding, the seed was washed with deionized water before being placed in the vial. The seeded vial was then transferred to a Crystalline multi-reactor equipped with a camera and laser. Transmissivity data from the laser were employed to calculate delay times (section 2.3.2), while images taken every 2 seconds were used to estimate crystal counts and secondary nucleation rates (section 2.3.3).

To investigate the influence of seed polymorphism on secondary nucleation kinetics, seeded crystallization with either α- or γ-glycine was performed at 25 °C and concentrations of 295, 310 and 320 mg g^−1^. However, since the previously reported faster growth rate of α-glycine could lead to the predominance of this solid form in the resulting crystalline product, additional experiments with γ-glycine seeds under conditions where the growth rate magnitudes of both solid forms were comparable were also conducted^42^ (320 mg g^−1^ and 20 °C).

Supersaturation values under these conditions are reported in Table S3 in the SI (section S2). To calculate the values of supersaturation, the solubilities of α- and γ-glycine at 20 and 25 °C were obtained from Manson *et al.*^41^ and converted to units of mg glycine per g of water. Additionally, the seed-on-a-stick methodology described in this section was also meticulously refined by successive control experiments in order to isolate the impact of the seed from other nucleation artifacts, as described in detail in section S3 of the SI.

#### 2.3.1 Temperature profile

The temperature profile consisted of four stages: (I) dissolution, (II) cooling, (III) isothermal agitation and (IV) nucleation. In stage I, the solution was held at 70 °C and 100 rpm for 30 minutes to ensure complete dissolution. In stage II, the mixture was cooled to the isothermal temperature (25 or 20 °C) at a rate of −5 °C min^−1^. A single-seed of the respective solid form of glycine was then fixed at 4 mm distance from the impeller tip, and the agitation rate was increased to 700 rpm. Experiments performed at 25 °C had a holding period of 200 minutes, while those conducted at 20 °C were held for 100 minutes. Regardless of the duration of the holding period, in stage III, the solution was stirred isothermally at 700 rpm, and in stage IV, solid fines appeared, which caused a decrease in transmissivity and an increase in crystal count.

#### 2.3.2 Delay time

Nucleation events were detected by transmissivity measurements, which monitor the extent of crystallization or dissolution by assessing how much light passes through a sample.^46^ The Crystalline equipment has an in-built laser and detector (Fig. 1) to measure transmissivity. A value of 100% indicated a fully homogeneous solution, while the onset of crystallization was detected as the intercept between the linear fit over the decrease in transmissivity with a value of 100%, as shown in Fig. S3 in the SI (section S4). The nucleation delay time was defined as the interval between the seeding point (*t*_0_) and the nucleation detection time (*t*).

#### 2.3.3 Secondary nucleation rate

The camera of the Crystalline system (Fig. 1), coupled with Technobis' image analysis algorithm, was employed to translate graphical images into crystal number count data as a function of time. A previously published calibration with polystyrene spheres^47^ (eqn (1)) was used to convert the total number of particles identified in the images (*N*) into suspension number density (*N*_p_), expressed in the number of crystals per unit volume.

1 *N*_p_ = 3.98*N*^2^ + 134.33*N* + 10

The secondary nucleation rate (SNR), defined as the rate of increase in the number of crystals within a specified volume, was estimated as the linear slope of the suspension number density curve, making sure that the upper limit of the calibration (1.6 × 10^5^ # mL^−1^ min^−1^) was not surpassed, as shown in Fig. S11 in the SI (section S5). SNRs were estimated for seed-on-a-stick, control and unseeded configurations.

#### 2.3.4 Polymorphic outcome of seeded crystallization

The product from each seeded crystallization was isolated, air-dried and characterized using infrared (IR) spectroscopy. Different solid forms can be detected with this technique by comparing the absorption spectra of infrared light, which can indicate different vibrational modes in crystal lattices. For glycine, polymorphic forms were identified in the spectral region ranging from 850–1000 cm^−1^, with the α-form showing a peak at 908 cm^−1^ and the γ-form at 927 cm^−1^.^17^

### 2.4 Control and unseeded experiments

Control experiments were conducted to explore whether seed holder surfaces in contact with the solution could trigger primary heterogeneous nucleation. Each control followed the same temperature profile as the corresponding SOAS experiment. However, upon reaching the isothermal temperature (20 or 25 °C), a capillary tube without a seed and with glue at the tip was introduced in the solution. Potential nucleation sites included the capillary tube, the glue, the Crystalline cap, and the overhead stirrer.

Unseeded experiments were performed to assess the potential contribution of primary nucleation in the vial. Unlike control experiments, the vial cap remained closed at all times as no capillary tube was introduced upon reaching the isothermal temperature. Instead, the solution was left undisturbed in the Crystalline vial while maintaining an agitation rate of 700 rpm, ensuring that no external surfaces interacted with the solution.

#### 2.4.1 Induction time

In this study, delay times refer exclusively to seeded crystallization, while induction times correspond to unseeded conditions. In both cases, the crystal detection time (*t*) was defined as the intercept of the linear fit over the decrease in transmissivity and a value of 100%, as explained previously in section 2.3.2. However, in control experiments the starting time (*t*_0_) was defined as the moment when the capillary tube was introduced in the solution, whereas for unseeded experiments, *t*_0_ corresponded to the time when the isothermal temperature was reached. Induction times were also calculated as the difference between *t* and *t*_0_ for all experiments.

### 2.5 Probability distribution of delay and induction times

Once delay times from seeded crystallization and induction times from control and unseeded experiments were determined, their probability distributions were calculated using eqn (2). In this equation, the probability (*P*(*t*)) is defined as the ratio of the cumulative number of experiments where nucleation occurred (*M*^+^) to the total number of experiments conducted (*M*).2
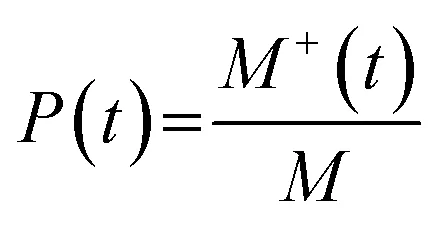


As shown in Tables S1 and S2 in the SI, 3–7 runs were performed for seeded and control experiments under each condition, while 25 unseeded experiments were conducted in each case. Therefore, a probability of 1 corresponds to 100% of experiments nucleating within the holding period where the solution was being isothermally agitated.

## 3 Results

### 3.1 Secondary nucleation kinetics are unaffected by the solid form of the seed

Seed-on-a-stick experiments with either α- or γ-glycine seeds were conducted at 25 °C to investigate the effect of seed polymorphism on secondary nucleation kinetics. The impact of seeding was assessed across a range of concentrations (295, 310 and 320 mg g^−1^) by comparing the probability distributions of delay and induction times (section 3.1.1). Additionally, secondary nucleation rates (SNRs) from seed-on-a-stick crystallization were then compared to SNRs obtained from control experiments without the seed which are due to spontaneous crystallization in the absence of externally introduced seed crystals (section 3.1.2).

#### 3.1.1 Probability distribution of delay and induction times

**Fig. 2:**
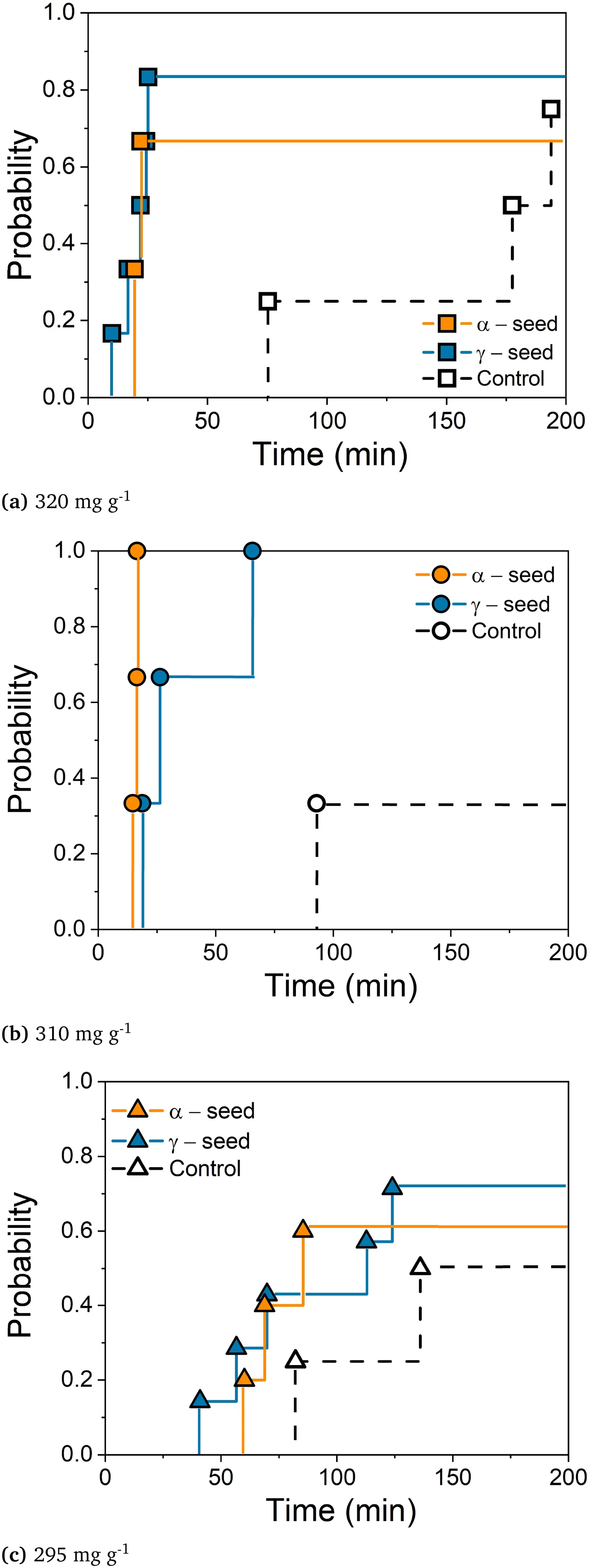
Probability distribution of delay and induction times at 25 °C for different concentrations: (a) 295 mg g^−1^, (b) 310 and (c) 320 mg g^−1^. Closed orange symbols represent delay times obtained with α-glycine. Closed blue symbols correspond to those obtained with γ-glycine seeds. Open black symbols indicate induction times from control experiments. The concentration is denoted by shape in all cases: triangles (△) for 295 mg g^−1^, circles (○) for 310 mg g^−1^ and squares (□) for 320 mg g^−1^. None of the unseeded experiments resulted in crystals within the 200 minute holding period.

The probability distributions calculated according to eqn (2), considered all crystallization trials performed at 25 °C, regardless of whether nucleation occurred or not. Table S1 in the SI shows the total number of experiments of each kind performed and the number which nucleated within a 200 minute holding period, and the detailed probability distributions are shown in Fig. 2.

In this figure, delay times from seeded crystallization with both α- and γ-glycine are significantly shorter than induction times from corresponding control experiments. These results confirm that both α- and γ-glycine seeds accelerated crystallization compared to control experiments, which take into account effects related to introducing the capillary tube used to hold the seed crystal. However, the seed solid form does not appear to have a significant impact on the probability distribution of delay times under these conditions.

At the lowest concentration tested (295 mg g^−1^; Fig. 2C), delay times from seeded experiments are closer to induction times from controls compared to experiments at higher concentrations. This suggests that the effect of the seed is diminished at lower supersaturation levels. In other words, as the concentration is reduced, it is more challenging to isolate and analyse the influence of the seed.

We note that none of the unseeded experiments performed at 25 °C crystallized within 200 minutes, which implies that the seed-on-a-stick experimental set-up itself promotes earlier nucleation – most likely due to heterogeneous nucleation on external surfaces (*e.g.*, Crystalline cap, capillary tube, or stirrer) or inadvertent self-seeding during cap changes.

Therefore, well-designed control experiments are indeed essential to assess the influence of the seed and to verify that crystallization is primarily driven by secondary nucleation mechanisms. Additional control experiments that helped refine the seed-on-a-stick methodology to minimize these experimental artifacts are presented in section S3 of the SI.

#### 3.1.2 Secondary nucleation rates

**Fig. 3:**
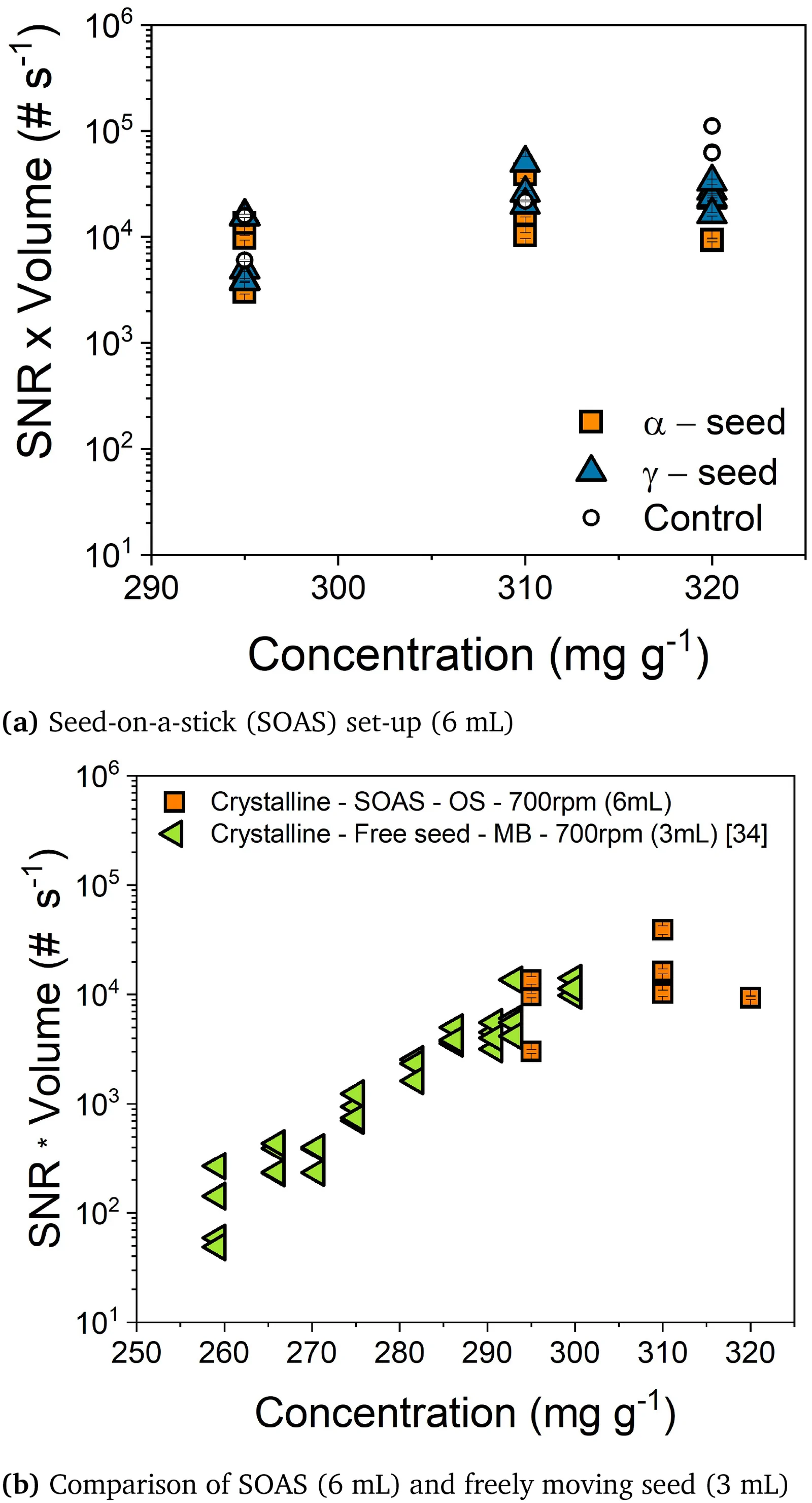
Secondary nucleation rates (SNRs) independent of volume as a function of concentration at an isothermal temperature of 25 °C. a) SNRs obtained in this study using a seed-on-a-stick (SOAS) configuration (6 mL). Closed orange symbols represent experiments seeded with α-glycine. Closed blue symbols indicate experiments seeded with γ-glycine seeds. Open symbols correspond to control experiments. b) Comparison of SNRs obtained in this work for α-glycine using a SOAS configuration (6 mL) with literature data for a single freely moving α-glycine seed crystal in a magnetically agitated vial (3 mL). Closed green triangles represent SNRs obtained from experiments in the Crystalline with a single α-glycine seed freely moving in solution at 3 mL volume and agitated with a PTFE magnetic bar.^34^

Once we established experimental conditions where glycine seeds accelerated crystallization compared to control experiments, secondary nucleation rates (SNRs) from seeded crystallization with both solid forms were compared to those obtained from control experiments without the seed, as shown in Fig. 3a. In this figure, SNRs from seeded crystallization with both α- and γ-glycine seeds exhibited similar trends, ranging from 1000 to 100 000 crystals per second, indicating that the solid form of the seed did not have a substantial effect on the magnitude of the nucleation rate measured by *in situ* imaging in the bulk solution.

Interestingly, however, similar SNRs were also measured from control experiments, which can be explained by a single nucleus mechanism, where a single crystal forms spontaneously *via* primary nucleation and subsequently grows and triggers secondary nucleation, effectively acting as an internal seed.

This interpretation was previously proposed by Cashmore *et al.*,^34^ who observed comparable secondary nucleation rates in seeded and unseeded systems of α-glycine under magnetic agitation. In that study, it was estimated that a single crystal spontaneously formed in the vial had to grow to a size larger than about 150 μm to initiate secondary nucleation and effectively act as a seed. It was concluded that in systems where secondary nucleation is much faster than primary nucleation, the overall SNR becomes independent of how the initial seed is introduced. Such a scenario is also relevant in this study, where the probability distribution of induction times indicates that primary nucleation is slow, producing on average one crystal every 10–100 minutes.

Additionally, SNRs obtained in this study with a seed-on-a-stick configuration with α-glycine were also compared with previously reported rates measured using single-crystal seeding in a magnetically agitated vial,^34^ as shown in Fig. 3b. In that work, a single α-glycine crystal was inserted into a vial where it was freely moving, so the effects of fluid shear and mechanical impact could not be isolated. To enable a direct comparison between the two studies, secondary nucleation rates were scaled by operating volume, normalising the SNRs to reflect the production rate of secondary nuclei per single crystal seed.

Using this approach, SNRs obtained with a seed-on-a-stick configuration at 6 mL with an overhead stirrer are consistent with those previously reported for a freely moving seed in a magnetically agitated vial at 3 mL.^34^ The comparable secondary nucleation rates observed in these two configurations indicate that fluid shear alone is sufficient to trigger secondary nucleation. Moreover, the similarity in rates suggests that mechanical impact, while present in magnetically agitated systems, is not a prerequisite for achieving secondary nucleation rates of the magnitude measured here.

### 3.2 Seeding with γ-glycine fails to suppress α-glycine crystallization

In the SOAS experiments presented above with either α- or γ-glycine seeds, the solid form of the seed was characterized using Raman spectroscopy (section 2.2), while the product from each crystallization was filtered and analyzed by IR spectroscopy (section 2.3.4).

Fig. S2 and S3 in the SI show the measured spectra of both the seed and product (solid lines) compared to the reference spectra of both solid forms of glycine (dashed lines). These data confirmed that the crystals produced in the presence of a single glycine seed were α-glycine in all cases regardless of the seed solid form, as a peak at 908 cm^−1^ was consistently measured in the IR spectra.

However, concomitant nucleation of both solid forms cannot be ruled out, as the relative growth rates of α- and γ-glycine at 25 °C are not known. It is possible that both solid forms nucleated, but α-glycine dominated due to a faster growth rate. Therefore, to further investigate secondary cross-nucleation events, SOAS experiments with γ-glycine seeds were conducted under conditions where the growth rate magnitudes of both solid forms become comparable as reported in the previous literature.^42^

#### 3.2.1 Secondary cross-nucleation with γ-glycine seeds

Additional seed-on-a-stick (SOAS), control, and unseeded experiments were conducted at 20 °C and 320 mg g^−1^, conditions under which the growth rate of γ-glycine is known to be relatively high (30 μm min^−1^) and similar to the growth rate of α-glycine.^42^ At this temperature, the solubility of γ-glycine is reported to be 205.7 mg g^−1^,^41^ resulting in the corresponding supersaturation ratio of 1.56.

**Fig. 4:**
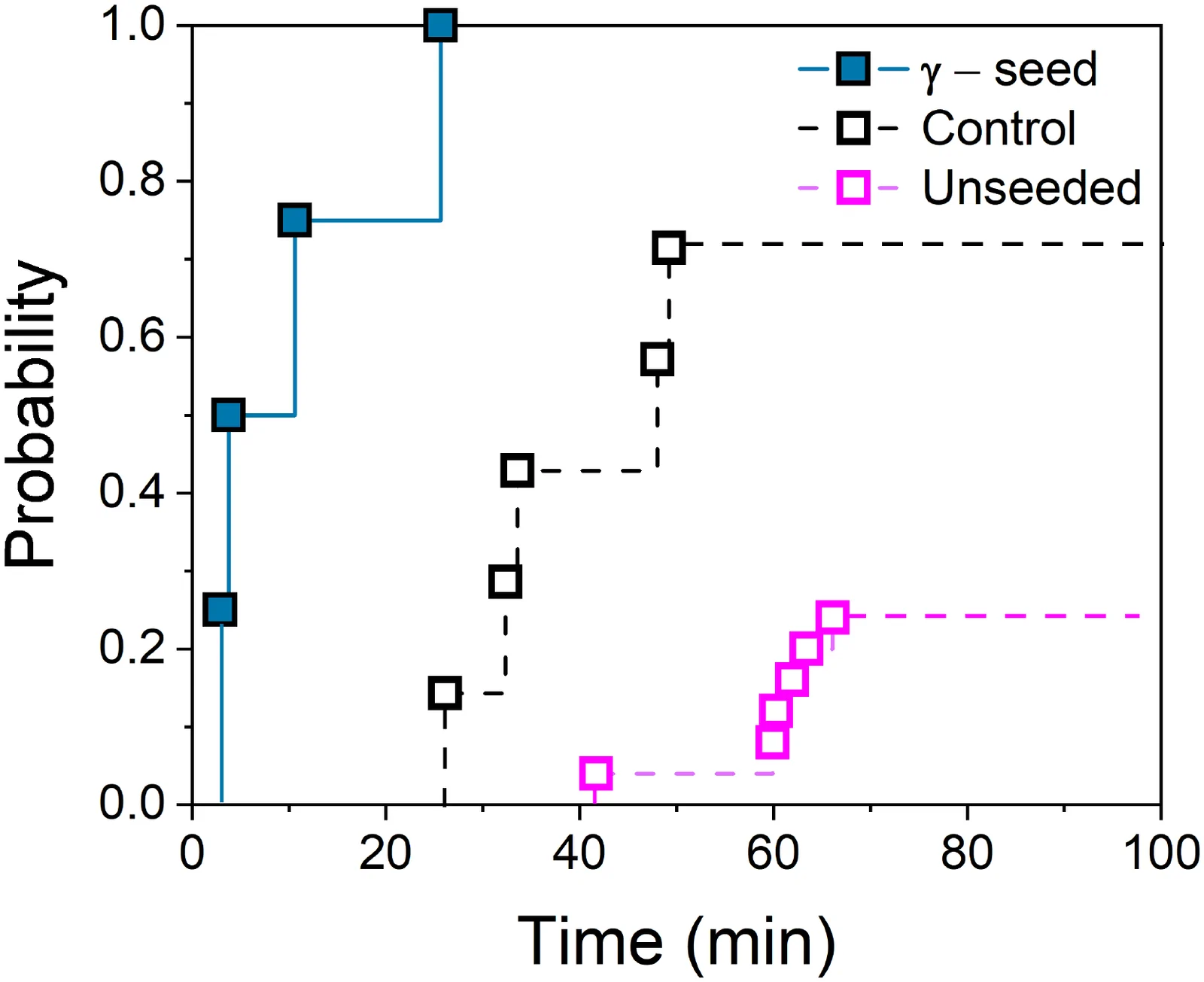
Probability distribution of delay and induction times at 320 mg g^−1^ and 20 °C. Closed blue squares indicate delay times from seeded crystallization with γ-glycine, open black squares correspond to induction times from control experiments and open purple squares represent induction times from unseeded experiments.

Due to this relatively high supersaturation, unseeded and control experiments were particularly important in this case to verify that primary nucleation was not the dominant crystallization pathway. Therefore, to assess the influence of seeding, probability distributions of delay and induction times were obtained under these conditions. The probability distributions are shown in Fig. 4, and Table S2 in the SI summarizes the number of successful nucleation events for each experimental setup at 20 °C.

This figure shows that introducing a γ-glycine seed again resulted in earlier nucleation when compared to control and unseeded experiments at 20 °C. This is evidenced by the shorter delay times and narrower probability distribution observed in the SOAS experiments. Similarly to experiments at 25 °C, the comparison between induction times in unseeded and control experiments indicates that the seed-on-a-stick experimental set-up itself promotes earlier nucleation, likely due to the presence of additional surfaces or unintended self-seeding during cap changes. Despite this, the clear separation between the distributions of delay and induction times confirms that the presence of the seed induces secondary nucleation under conditions of higher supersaturation and seed growth.

**Fig. 5:**
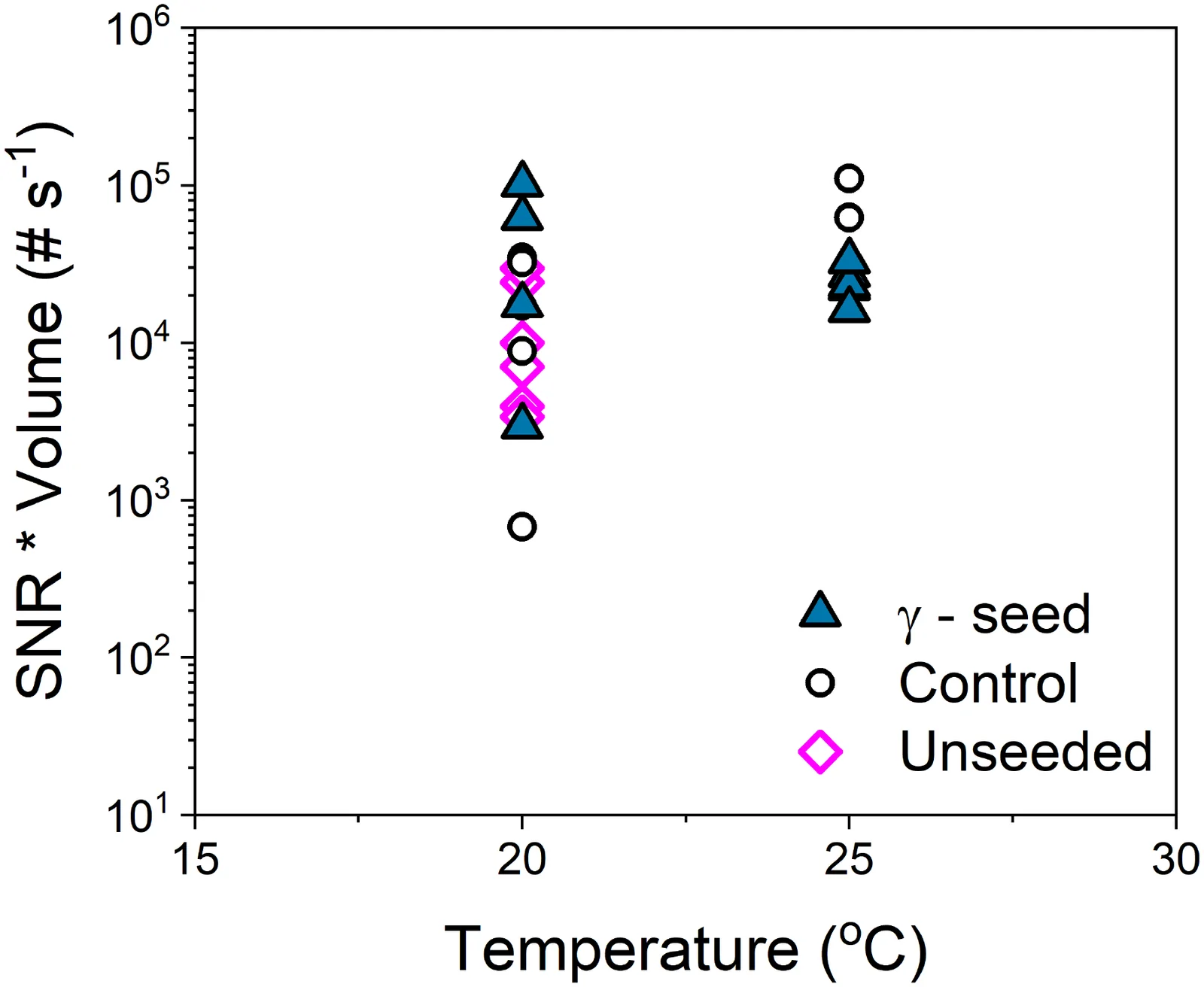
Secondary nucleation rates from seeded, control and unseeded experiments at 320 mg g^−1^. Closed blue triangles indicate seeded crystallization with γ-glycine, open black circles correspond to control experiments and open purple diamonds represent unseeded experiments.

We also determined the secondary nucleation rates (SNRs) from these experiments at 20 °C and compared them with the values reported above at 25 °C at the same concentration (section 3.1.2), as shown in Fig. 5.

Secondary-cross nucleation phenomena were confirmed in single-seed crystallization by comparing the polymorphism of the initial seed to that of the resultant product. In all seeded crystallization with γ-glycine at 20 °C, IR spectroscopy confirmed that the resulting crystals were of the α-form. These results are consistent with the findings at 25 °C using γ-glycine seeds, as shown in Fig. S3 in the SI. Therefore, α-glycine secondary nuclei consistently formed, even under conditions where a low growth of γ-glycine could be ruled out.

## 4 Discussion

In this study, we demonstrate that secondary nucleation can be induced solely by the effect of fluid shear in the absence of mechanical impact, and can be isolated from other sources of nucleation at a small scale (6 mL). While the presence of a single seed crystal on a stick induced earlier nucleation of glycine compared to control experiments, the seed presence or its solid form did not have significant influence on either secondary nucleation kinetics or the solid form of the resulting crystalline product.

### 4.1 Evidence of secondary nucleation induced by fluid shear

The role of fluid shear in inducing secondary nucleation has been a subject of debate in the literature. Traditionally, secondary nucleation has been attributed mainly to attrition and particle fragmentation processes. This view was largely shaped by early studies employing relatively large inorganic seed crystals suspended in non-solvents^48,49^ or saturated solutions^50,51^ in order to suppress growth or nucleation and isolate mechanical impact as the dominant mechanism.

However, some studies using smaller organic crystals have challenged this perspective. For instance, Souza *et al.*^52^ reported that paracetamol crystals (355 μm to 500 μm) suspended in a low solubility solvent like cyclohexane exhibited no detectable increase in FBRM counts for more than four hours at 375 rpm. However, seeded crystallization of paracetamol in 2-propanol solutions under the same agitation conditions produced an immediate and substantial increase in particle counts right after seeding, indicating that secondary nucleation was not governed by attrition.

Nevertheless, it remained possible that the rapid increase in crystal counts observed upon seeding could arise from initial breeding or mechanical contact. To assess a potential contribution of mechanical impact, Tyrrell *et al.*^53^ employed high-velocity fluid-jet apparatus coupled with shadowgraphy to quantify particle–wall collisions of small paracetamol crystals (100 μm to 400 μm). In that study, they reported no evidence of breakage with particle velocities of up to 10 m s^−1^ as well as negligible collisions for particles below 300 μm, suggesting that fluid shear effects may play a more significant role than was previously assumed.

To isolate the effect of fluid shear on secondary nucleation, single fixed-seed crystallization techniques (“seed-on-a-stick” experiments) have been employed across various experimental configurations.^24–26,38,39,54,55^ However, as recently emphasised by De Vrieze and Kuhn,^40^ many of these studies lacked rigorous control experiments, making it difficult to determine whether earlier nucleation – or the formation of different chiral forms to that of the seed – was genuinely induced by the seed crystal rather than by other introduced surfaces such as seed holders. Their systematic replication work demonstrated that, when appropriate control experiments are implemented, distinguishing fluid shear driven secondary nucleation from primary nucleation (spontaneously occurring in the absence of pre-existing crystals) is less straightforward than commonly assumed.

The present study reinforces the importance of control experiments for isolating fluid shear driven secondary nucleation. Using a seed-on-a-stick configuration, we demonstrated that nucleation occurred earlier in the presence of a seed crystal in comparison with the corresponding control experiments without the seed. However, this distinction was only evident after a systematic refinement of the seed-on-a-stick methodology (section S3 in the SI), which aimed to eliminate unintended nucleation arising from the introduction of external surfaces. Once these artefacts were removed and the influence of initial breeding was avoided by slightly dissolving the seed crystal with deionised water, delay times from seeded crystallization were consistently shorter than induction times from the respective control experiments under all conditions investigated in this work.

### 4.2 Secondary nucleation rates were unaffected by the seed presence or its solid form

Although the introduction of a glycine seed crystal, irrespective of its solid form, led to earlier nucleation relative to the control experiments, secondary nucleation rates (SNRs) remained unchanged. In fact, SNRs obtained from seed-on-a-stick experiments with both α- and γ-glycine seeds were similar to those measured in control and unseeded crystallization experiments.

This observation is consistent with a previous report on glycine crystallization from aqueous solutions by Cashmore *et al.*^34^ In that study, a single α-glycine crystal was inserted into a magnetically agitated vial – rather than fixed in a seed-on-a-stick configuration – and secondary nucleation rates were compared to those measured in unseeded crystallization. Using a particle-counting methodology similar to the one employed in this work, it was found that seeded and unseeded systems exhibited comparable secondary nucleation rates. In that work, primary nucleation rates (PNRs) were also estimated from the probability distribution of induction times, assuming a stochastic nucleation process.^56^ When these PNRs were compared to SNRs measured with a particle-counting technique, SNRs were found to be 3–4 orders of magnitude higher.

The disparity between primary and secondary nucleation rates observed in this system can provide an explanation for why unseeded crystallization exhibits longer induction times but ultimately displays similar SNRs to seeded crystallization. In the unseeded case, it takes longer for the first primary nucleus to form, which is an inherently slow and stochastic process because of the low PNR. However, once the first crystal emerges and grows sufficiently large, the much faster secondary nucleation process dominates and dictates the measured (secondary) nucleation rate.

Consequently, whether secondary nuclei originate from a deliberately introduced fixed seed crystal – as in a seed-on-a-stick configuration – or from a crystal grown from the first primary nucleus in an unseeded system, the resulting SNRs remain similar. The presence of a seed crystal merely eliminates the stochastic delay associated with the first primary nucleus formation event; it does not alter the resulting rate of secondary nucleation, which is mainly dictated by solute concentration and temperature as well as fluid dynamic conditions in each experiment.

### 4.3 Consistent formation of α-glycine in seeded and unseeded systems

In this study, the presence or the solid form of the seed had no measurable effect on the polymorphic outcome of the resulting crystalline product. Seeding with γ-glycine resulted in the formation of α-glycine which was also observed in control and unseeded experiments. This aligns with previous observations in unseeded aqueous glycine systems, where agitation favoured the formation of the metastable α-form.

As shown by Vesga *et al.*,^17^ in unseeded crystallization of glycine from aqueous solutions, γ-glycine can arise directly from quiescent solutions at high concentrations, whereas under agitation, α-glycine was consistently obtained. In that study, the preference for α-glycine under flow conditions was attributed to shear-induced modifications of mesoscale clusters in solution. Therefore, the predominance of α-glycine in our unseeded and control experiments can be expected. In a study on effects of the shear rate and glass-solution interfacial area on primary nucleation kinetics in aqueous glycine solutions,^57^ it was found that the primary nucleation rate was proportional to both the shear rate and the glass-solution surface area, and it was suggested that shear-induced and surface-assisted aggregation of mesoscale clusters appears to facilitate glycine nucleation.

The persistence of α-glycine – irrespective of the presence or the solid form of the seed – suggests that fluid shear induced secondary nucleation may work in a manner similar to primary nucleation induced by cooperative effects of fluid shear and the solution–solid interface. We note that the introduction of external surfaces, such as seed holders in our control experiments or PTFE coated magnetic stirrers in previous work by Vesga *et al.*,^17^ led to earlier nucleation compared to unseeded experiments, which suggests that interface induced nucleation is important in this system. Yet, the presence of a glycine seed crystal, irrespective of its solid form, resulted in even earlier nucleation (as evidenced by shorter delay times) than in the corresponding control experiments. This suggests that interface induced effects on nucleation can be even more effective when the surface and solute share the same chemical identity, irrespective of the surface solid form.

### 4.4 Nucleation in the interfacial solution layer as a unifying mechanism for shear-induced primary nucleation, secondary nucleation and cross-nucleation

A plausible mechanism unifying these observations may be that mesoscale precursor clusters responsible for the formation of α-glycine in unseeded systems are present in the interfacial solution layer, including the vicinity of a seed crystal. Interactions of these clusters with the surface and fluid shear at the interface then influence their stability and further aggregation and the resulting likelihood of an initial nucleus formation.

Both the ECSN^27^ and SNIPE^28–30^ models offer plausible theoretical frameworks for the unifying mechanism proposed above. In the ECSN model, solute clusters responsible for the formation of the α-form may be attracted by van der Waals interactions to the interfacial solution layer at the surface of any solid in contact with the solution, including an α-glycine or a γ-glycine seed, where they aggregate to form crystal nuclei. Alternatively, in the SNIPE model, α-form nuclei may form preferentially within an interfacial solution layer, where the local energy barrier for nucleation is reduced, depending on the van der Waals interactions with the surface. In both of these cases, there is no intrinsic difference between primary nucleation (in the absence of pre-existing crystals) and secondary nucleation (in the presence of crystals of the same chemical identity as the solute but of any solid form), apart from different strengths of van der Waals interactions of the surface with solution components. In this mechanism, primary nucleation would be surface-induced but distinct from the classical heterogeneous primary nucleation where the crystal nucleus is assumed to be in physical contact with the surface. Furthermore, in the case of secondary nucleation, the solid form of the resulting nuclei may depend just on its chemical composition, and not necessarily on the seed crystal polymorph, resulting in potential cross-nucleation.

Fluid flow can play multiple roles in this unifying mechanism, including deformation or aggregation of mesoscale clusters and transport of clusters or nuclei between the interfacial solution layer and the bulk solution. Further studies of how fluid shear and different surfaces, including those of pre-existing crystals, act together to impact nucleation from solutions will be critical for better understanding of nucleation kinetics and developing predictive modelling tools for designing and scaling up crystallization processes.

## 5 Conclusions

This study investigated shear-induced secondary nucleation of glycine in aqueous solutions. Secondary nucleation was assessed through delay and induction times for seeded and control/unseeded experiments, respectively, and *in situ* secondary nucleation rate measurements. The comparison of unseeded and control experiments highlighted the importance of diligently executed control experiments to differentiate between secondary and primary nucleation as well as experimental artifacts.

Our carefully conducted experiments showed that the presence of seed crystals resulted in earlier nucleation compared to control and unseeded experiments, demonstrating that secondary nucleation can be induced solely by the effect of fluid shear in the absence of mechanical impact. Notably, the solid form of the seed crystal had no impact on either measured secondary nucleation rates or the solid form of the crystals produced. The metastable α-form consistently formed, regardless of the seed solid form, confirming fluid-shear induced cross-nucleation with γ-glycine seeds.

We proposed a unifying mechanism for shear-induced primary nucleation, secondary nucleation and cross-nucleation, based on mesoscale precursor clusters responsible for the formation of crystal nuclei, where van der Waals interactions of these clusters with surfaces in contact with the solution influence their stability and aggregation resulting in crystal nuclei formation. Fluid flow can induce deformation or aggregation of mesoscale clusters and transport of clusters or nuclei between the interfacial solution layer and the bulk solution. In this mechanism, there is no intrinsic difference between primary and secondary nucleation. Primary nucleation would be surface-induced but distinct from the classical heterogeneous primary nucleation, while for secondary nucleation, the surface effect may depend just on its chemical composition, and not necessarily on its solid form.

Further research on how fluid shear and different surfaces, including those of pre-existing crystals, act in concert to influence nucleation will be crucial for deeper understanding of nucleation kinetics and developing predictive modelling tools for designing and scaling up of crystallization processes.

## Conflicts of interest

There are no conflicts to declare.

## Supplementary Material

CE-028-D6CE00279J-s001

## Data Availability

The data supporting this article have been included as part of the supplementary information (SI). Supplementary information: contains spectral characterization of seed crystals and products, a summary of experiments performed and corresponding supersaturation ratios, and additional control experiments. Representative examples of delay and induction time measurements and secondary nucleation rate estimations are also provided. See DOI: https://doi.org/10.1039/d6ce00279j.
