# Supplementary material for: Fluid shear driven secondary nucleation using α-and γ-glycine seeds confirmed with rigorous control experiments
Source: CrystEngComm. 2026 Apr 22;28(22):3475–85. doi: 10.1039/d6ce00279j (PMC13584634; doi:10.1039/d6ce00279j)
Supplement: Supplementary file 1 [file CE-028-D6CE00279J-s001.pdf]

Supplementary Information (SI) for CrystEngComm.
This journal is © The Royal Society of Chemistry 2026

Supplementary Information

Fluid-Shear Driven Secondary Nucleation Using α- and γ-Glycine Seeds Confirmed with Rigorous Control Experiments

Lucas Nahas,*[a] Mei Lee,[b] Mark Haw,[a] and Jan Sefcik[a]

[a] *Department of Chemical and Process Engineering, University of Strathclyde, Glasgow, UK.*
[b] *Substance Development – Materials Science- Medicine Development & Supply, R&D, GSK, Stevenage, UK.*

Corresponding Author: Lucas Nahas, Email: lucas.nahas-martinez.2017@uni.strath.ac.uk

Table of Contents

Contents

S1 Spectral Characterization of Seeded Crystallizations with α- and γ-Glycine. 1

S2 Summary of Seeded, Control and Unseeded Experiments 2

S3 Refinement of the Seed-on-a-Stick Methodology via Control Experiments. 3
S3.1 Effect of Vial Sealing and Encrustation 3
S3.2 Effect of Capillary Tube Cleaning 5
S3.3 Effect of a Standardized Stirrer Washing Procedure 6
S3.4 Effect of Parafilm in Contact with the Solution 7
S3.5 Refined Methodology 8

S4 Delay and Induction Times Measured by Transmissivity 9

S5 Estimation of Secondary Nucleation Rates 10

S6 Suspension Number Density Profiles for Seed-on-a-Stick, Control, and Unseeded Experiments 11

S1 Spectral Characterization of Seeded Crystallizations with α- and γ-Glycine.

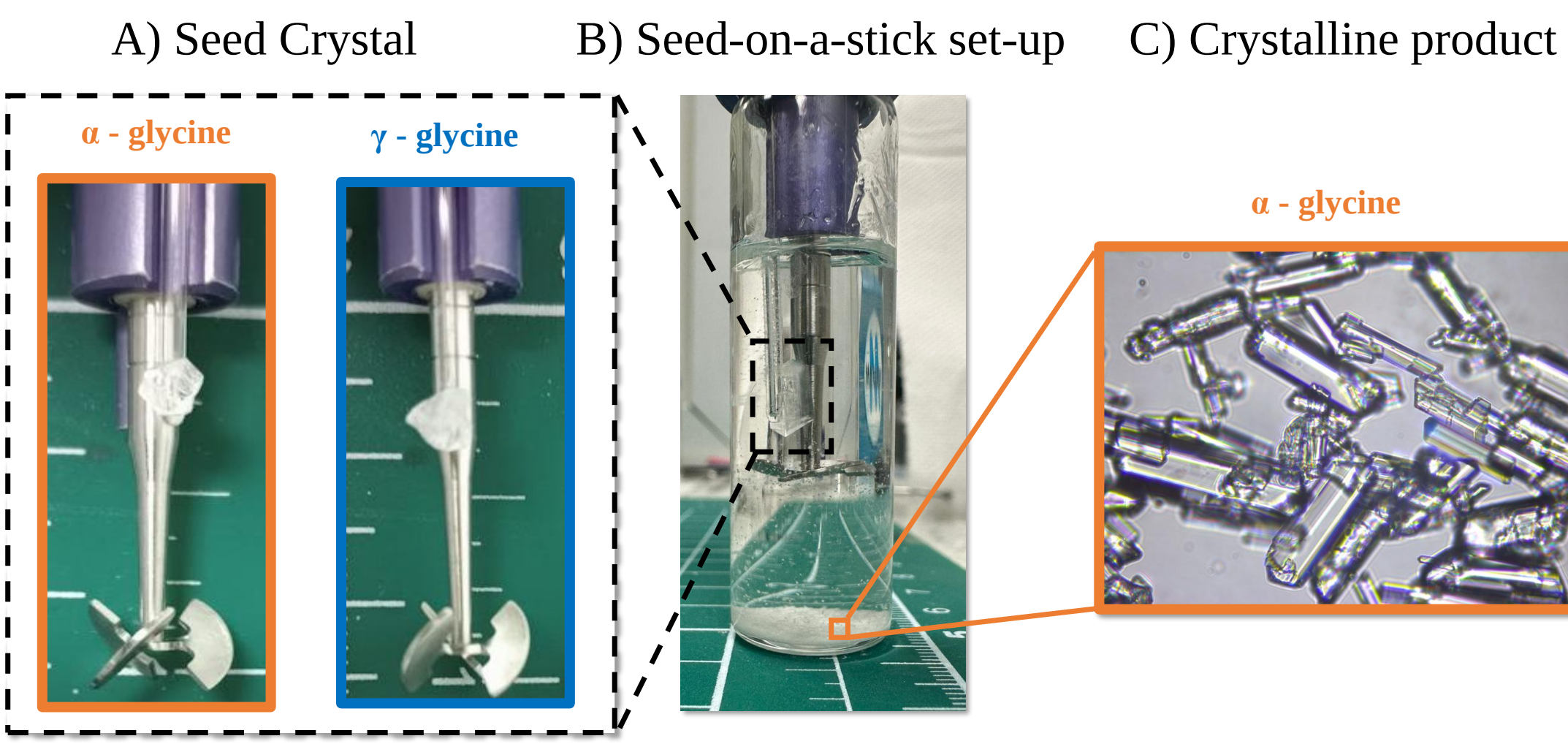

Figure 1 (a) Seed-on-a-stick configuration prior to the crystallization experiment. Images were taken before the washing step, as the seed had to be introduced directly into the vial immediately after being briefly dissolved with deionised water; thus, the apparent seed size corresponds to an estimate prior to washing. (b) Seed-on-a-stick set-up inside a Crystalline® vial after completion of a crystallization experiment, showing clear seed growth and accumulation of crystalline material at the bottom of the vial. (c) Microscope images of the resulting product slurry, showing the morphology and dispersion of the obtained crystals.

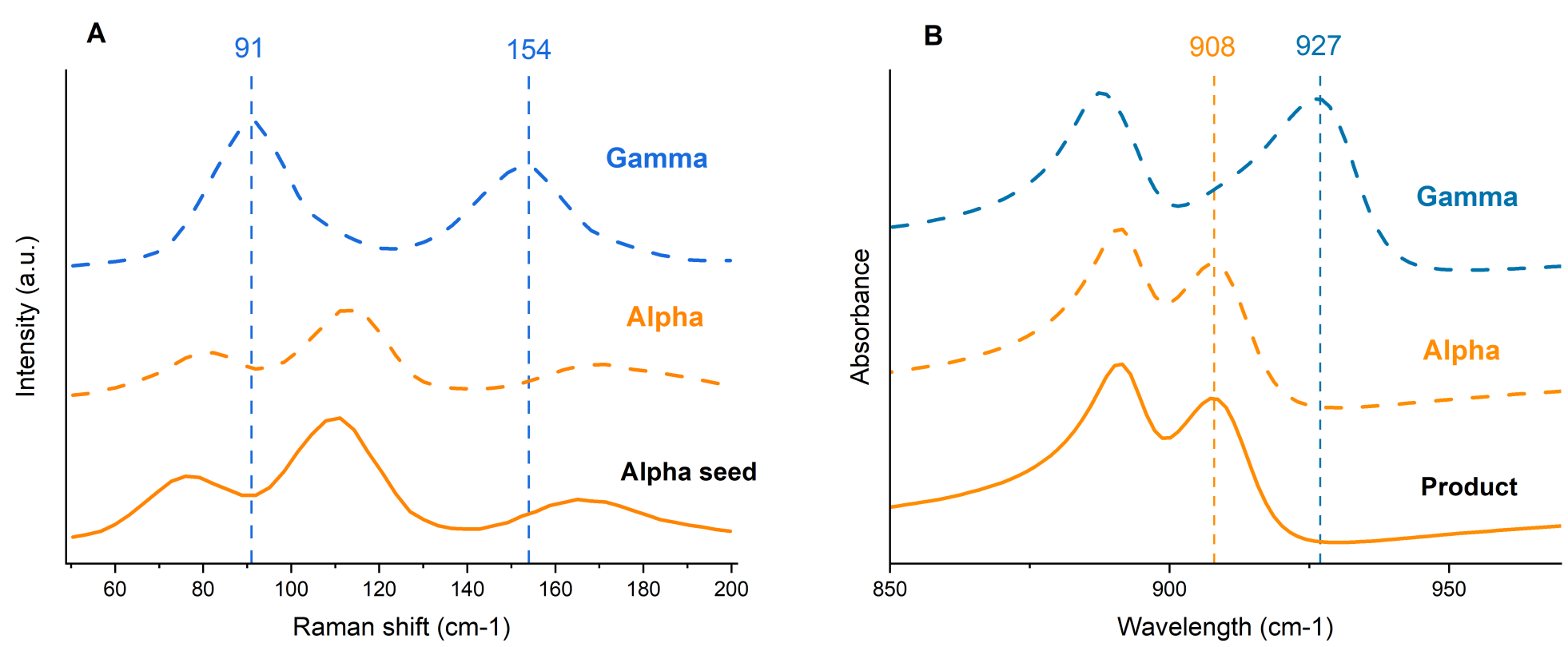

Figure 2 (A) Raman spectrum of an α-glycine seed and (B) IR spectrum of the product from a single-seed crystallization with α-glycine. Solid lines indicate measured spectra, while dashed lines represent reference spectra for both solid forms.

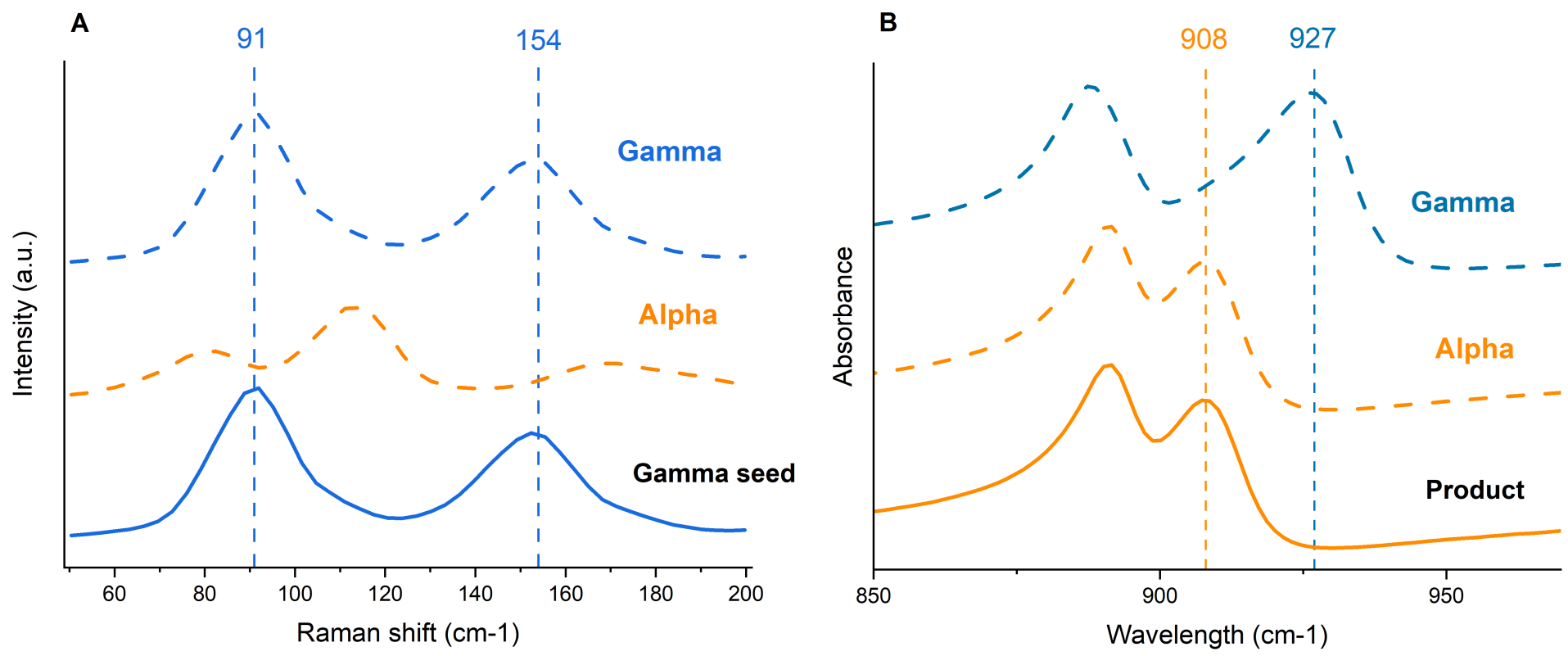

Figure 3 (A) Raman spectrum of a γ-glycine seed and (B) IR spectrum of the product from a single-seed crystallization with γ-glycine. Solid lines indicate measured spectra, while dashed lines represent reference spectra for both solid forms.

S2 Summary of Seeded, Control and Unseeded Experiments

Table 1 Summary of experiments conducted at 25 °C and nucleation outcomes.

Experiment Type	Number of runs	Nucleated
	320 mg/mL	
Seeded with γ	6	5
Seeded with α	3	2
Control	4	3
Unseeded	25	0
	310 mg/mL	
Seeded with γ	3	3
Seeded with α	3	3
Control	3	1
Unseeded	25	0
	295 mg/mL	
Seeded with γ	7	5
Seeded with α	5	3
Control	4	2
Unseeded	25	0

Table 2 Summary of experiments conducted at 20 °C and nucleation outcomes.

Experiment Type	Number of runs	Nucleated
	320 mg/mL	
Seeded with γ	4	4
Control	7	5
Unseeded	25	6

Table 3 Experimental conditions for seeded crystallisation experiments

Solution Concentration (mg g^{-1})	Isothermal Temperature (°C)	Seed Solid Form	Supersaturation
295	25	γ	1.29
		α	1.18
310	25	γ	1.35
		α	1.24
320	25	γ	1.40
		α	1.28
	20	γ	1.56

S3 Refinement of the Seed-on-a-Stick Methodology via Control Experiments.

In all seeded and control experiments presented in the main text, a refined seed-on-a-stick methodology was employed. This refinement was developed by analyzing induction times from control experiments that overlapped with delay times from seeded crystallizations, and identifying the factors that led to an early detection of nucleation in the controls. By iteratively adjusting the experimental protocol, we ensured that fluid-shear driven secondary nucleation induced by a single fixed crystal seed - instead of other nucleation artifacts - was the main mechanism driving crystallization in all the seeded runs. This optimized methodology involved four key steps: solution preparation, pre-heating of Crystalline vials, standardized stirrer washing protocol and capillary tube and seed cleaning.

For each concentration, solutions were prepared and dissolved externally, while four empty vials were pre-heated inside the Crystalline system at 70 °C for 15 minutes. Subsequently, a preheated pippete was used to transfer 6mL of the warm solution into each vial, which was immediately sealed with Crystalline caps containing an O-ring and a 3-blade short overhead stirrer. The solution was then agitated at 100 rpm for 30 minutes at 70 °C to dissolve any remaining crystals before cooling to the target isothermal temperature. Simultaneously, four additional vials, with 6mL of deionized water, were placed in parallel inside the Crystalline system and subjected to the same temperature and agitation profiles. These vials contained the overhead stirrers that would later be transferred to the supersaturated solution when isothermal conditions are reached.

This standardized stirrer washing procedure served two purposes: first, to eliminate any dust or residual contaminants that could result in unintended nucleation; and second, to thermally equilibrate the stirrers by subjecting them to the same temperature profile than the solution, minimizing any crystallization artifact caused by temperature fluctuations at the seeding point. Lastly, once the solution was cooled to the designated isothermal temperature (20 or 25 °C), the Crystalline caps equipped with the washed stirrers were assembled with capillary tubes that had a single crystal seed glued to the tip.

Before seeding, the fixed single crystal was slightly dissolved with deionized water to avoid initial breeding. Subsequently, the capillary tube with the washed seed was located at a distance of 4mm from the tip of the impeller and a change of caps was performed to introduce the seed in the supersaturated solution. After the seeding point, the solution was agitated at 700 rpm for 100 minutes at 20°C or 200 minutes at 25 °C. At this stage, the onset of crystallization was measured by a drop in transmissivity, which measures the amount of light that passes through a sample.

To obtain this refined methodology, however, control experiments without the seed were conducted only at the highest supersaturation level investigated in the main text (320 mg/mL and 20 °C). The aim was to examine the effect that small modifications to the experimental procedure would have on measured induction times.

S3.1 Effect of Vial Sealing and Encrustation

Initially, control experiments were performed without prior external dissolution of the solute. Instead, glycine crystals were weighed directly into each vial, followed by the addition of 6 mL of deionized water at ambient temperature. The vials were then fitted with Crystalline caps equipped with overhead stirrers, and the suspension was dissolved in-situ by stirring at 700rpm and heating to 60 °C at a rate of 5 °C per minute. After maintaining agitation at 700 rpm and 60 °C for 30 minutes, the dissolved solution was subsequently cooled to 20 °C at a rate of -5 °C per minute. Upon reaching the isothermal temperature, overhead stirrers were replaced by analogous Crystalline caps that were fitted with a capillary tube secured with a small amount of parafilm, as shown in Figure 4.

These stirrers, however, were not subjected to a standardized washing procedure, instead they were just washed with deionized water at ambient temperature. Additionally, O-rings were not used in this case, which are necessary to ensure a proper seal between the vial and the cap. Two types of controls were conducted with this setup: one in which a small amount of glue was applied at the tip, and another where only capillary tubes were used. The resulting probability distributions of induction times for both configurations are presented in Figure 4, alongside previously reported delay and induction times from seeded and unseeded experiments conducted under the same conditions with a refined methodology.

As shown in this figure, induction times from control experiments with this set-up overlapped with delay times from seeded crystallizations. This overlap was observed irrespective of whether capillary tubes were introduced with or without glue at the tip. Importantly, since none of the controls were subject to a standardized stirrer washing procedure, residual contaminants on the surface of the stirrer, as well as uncontrolled temperature fluctuations, could have contributed to the unexpectedly short induction times observed.

Due to the absence of an O-ring during the dissolution stage, the Crystalline cap was not properly sealed. This often resulted in solvent evaporation and the subsequent formation of a crystal crust on the internal - and occasionally external- surfaces of the vial.The formation of this crust is problematic, as a change of stirrers is required once isothermal conditions are reached, which can, in turn, also lead to inadvertent self-seeding.

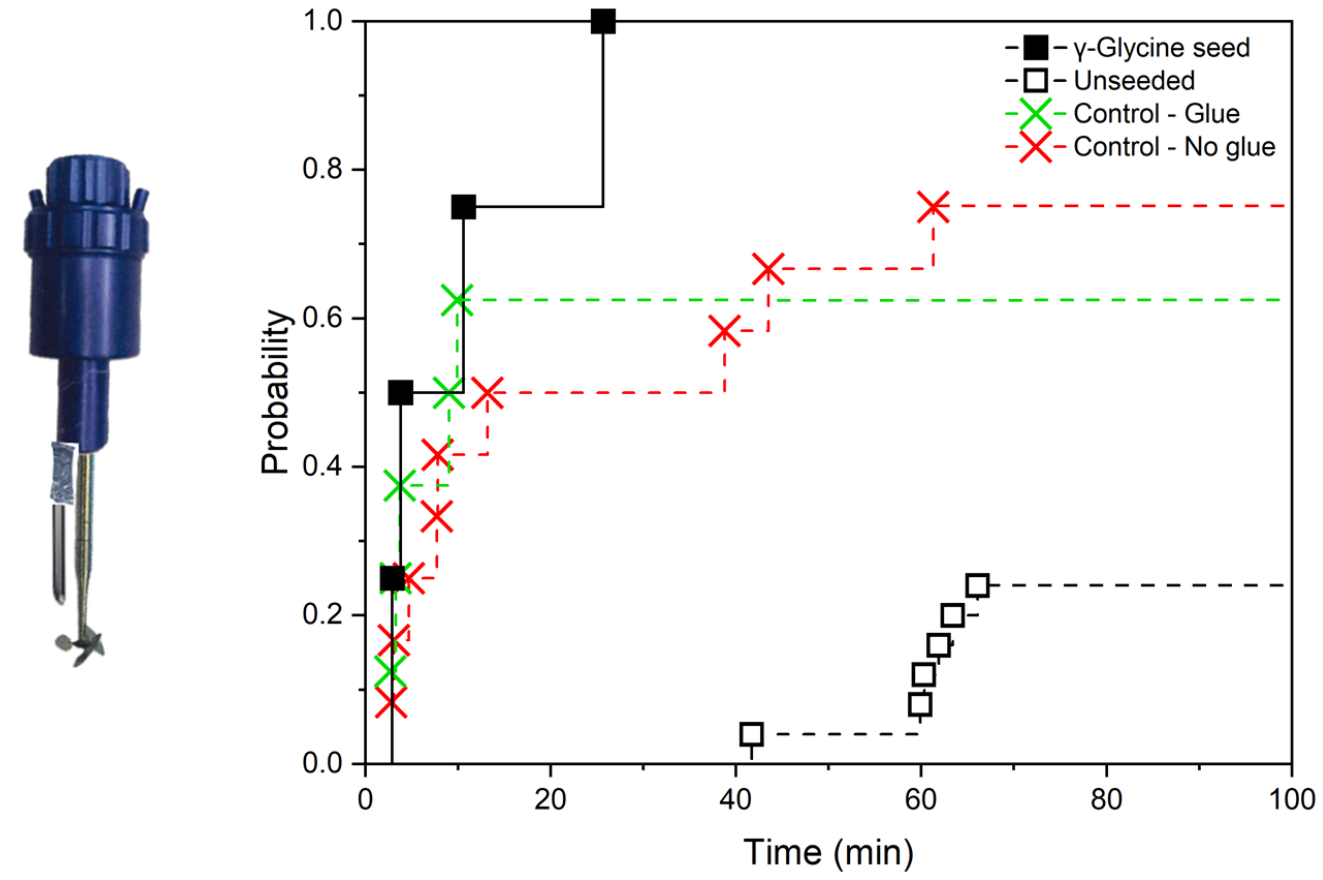

Figure 4 Probability distributions of induction times from control experiments conducted without an O-ring at 20 °C, 320 mg/mL, and 700 rpm. Green crosses represent control experiments in which a small amount of glue was applied to the tip of the capillary tube; red crosses indicate experiments using the capillary tube without glue. Black filled squares correspond to delay times from seeded crystallizations with γ-glycine, while black open squares indicate induction times from unseeded crystallizations under the same conditions.

Furthermore, it was found that even at a stirring speed of 700 rpm, overhead stirrers were unable to fully suspend the solute when it was added directly to the vial, resulting in the formation of an undissolved solute layer accumulating at the bottom in some cases. As a consequence, not all control experiments crystallized, which explains why the probability distributions of the controls did not reach 1 in the corresponding figure. In control experiments where crystallization did not occur, the solute remained undissolved due to inadequate suspension by the stirrer.

To address these issues, additional control experiments were conducted using vortex agitation before introducing the vial into the Crystalline system. An O-ring was also used to ensure proper sealing during the dissolution stage. The resulting probability distributions of induction times are shown in Figure 5 below.

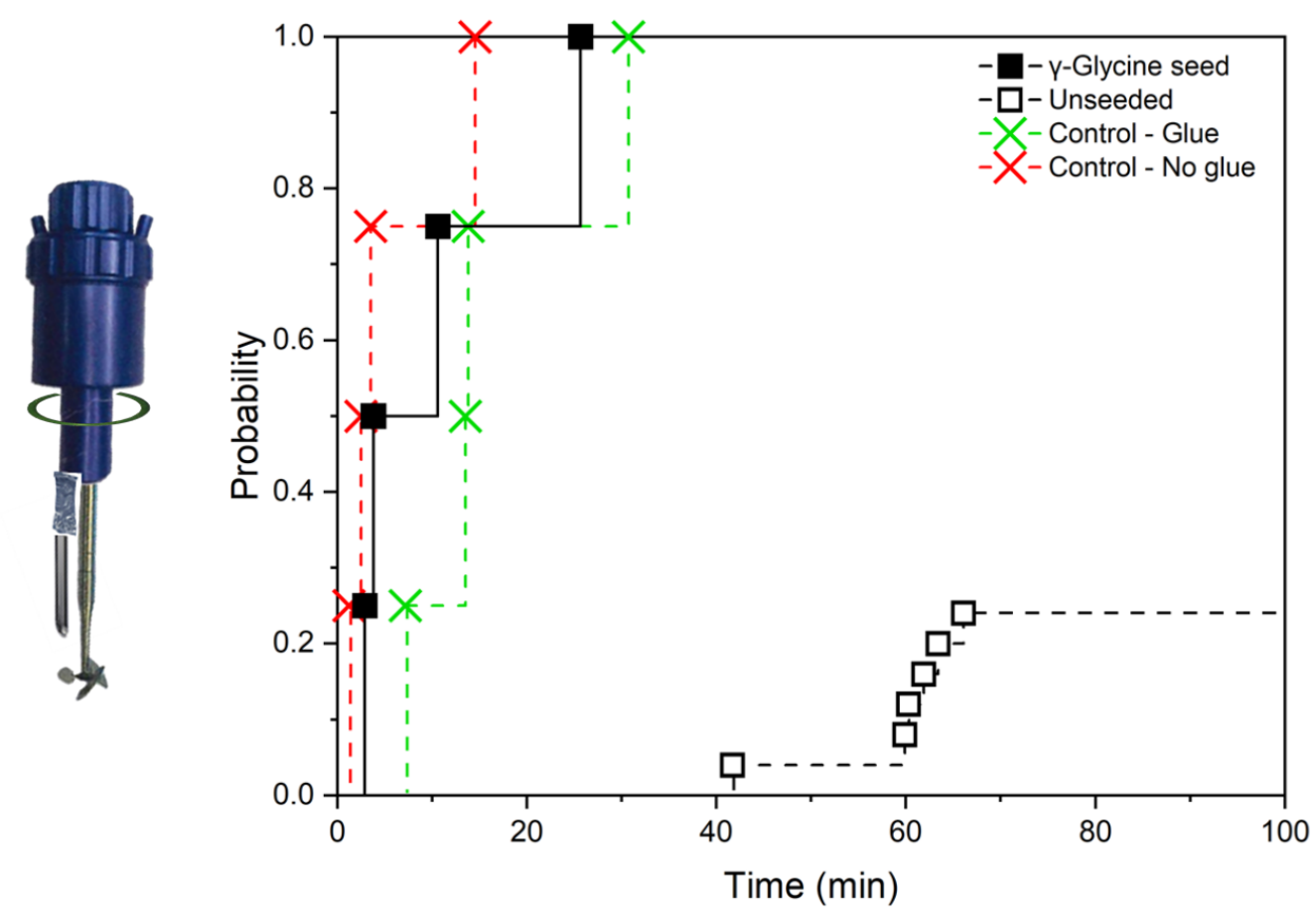

Figure 5 Probability distributions of induction times from control experiments conducted with an O-ring and vortex agitation at 20 °C, 320 mg/mL, and 700 rpm. Green crosses represent control experiments in which a small amount of glue was applied to the tip of the capillary tube; red crosses indicate experiments using the capillary tube without glue. Black filled squares correspond to delay times from seeded crystallizations with γ-glycine, while black open squares indicate induction times from unseeded crystallizations under the same conditions.

In this figure, the effect of prior vortex agitation to avoid the formation of a cake can be clearly observed, since 100 % of control experiments crystallized - whether glue was added at the tip of the capillary tube or not. However, even though solute settling was prevented by more uniform suspension, this approach also led to undissolved crystals adhering initially to the rim of the vial due to vortex agitation, which can later dislodge when the cap is replaced at the seeding point, resulting in unintended nucleation.

In this set-up, the use of an O-ring mitigated external encrustation, but a crystal crust still formed along the inner walls of the vial, just above the liquid level. Additionally, since no stirrer washing procedure was performed in these runs either, residual particles or differences in temperature may have also contributed to earlier crystallization. As a result of these nucleation artifacts, induction times from control experiments still overlapped significantly with delay times from seeded crystallizations, as shown in Figure 5.

S3.2 Effect of Capillary Tube Cleaning

To prevent internal encrustation while ensuring complete solute dissolution, control experiments were also performed where the bulk solution was prepared externally and transferred warm to the vials. With this set-up, empty vials were preheated within the Crystalline system at 70 °C for 15 minutes while the solution was being dissolved externally. Following thermal equilibration, 6 mL of warm solution were transferred into each vial using a pre-heated pipette. The vials were then sealed with Crystalline caps equipped with O-rings and overhead stirrers.

To mitigate internal encrustation, the stirring speed during the dissolution phase was reduced to 100 rpm, as rapid agitation was unnecessary due to prior external dissolution. The solution was then maintained at this agitation speed for 30 minutes at 70 °C - to ensure complete dissolution - before being cooled to 20 °C at a rate of -5 °C per minute. Upon reaching isothermal conditions, the overhead stirrers were replaced with analogous Crystalline caps, each fitted with a capillary tube containing glue at the tip and secured in place using parafilm. Additionally, for these controls, the effect of washing the capillary tubes with deionized water at different temperatures (17 °C and 40 °C) was also investigated and the detailed probability distributions of induction times are shown in Figure 6 below.

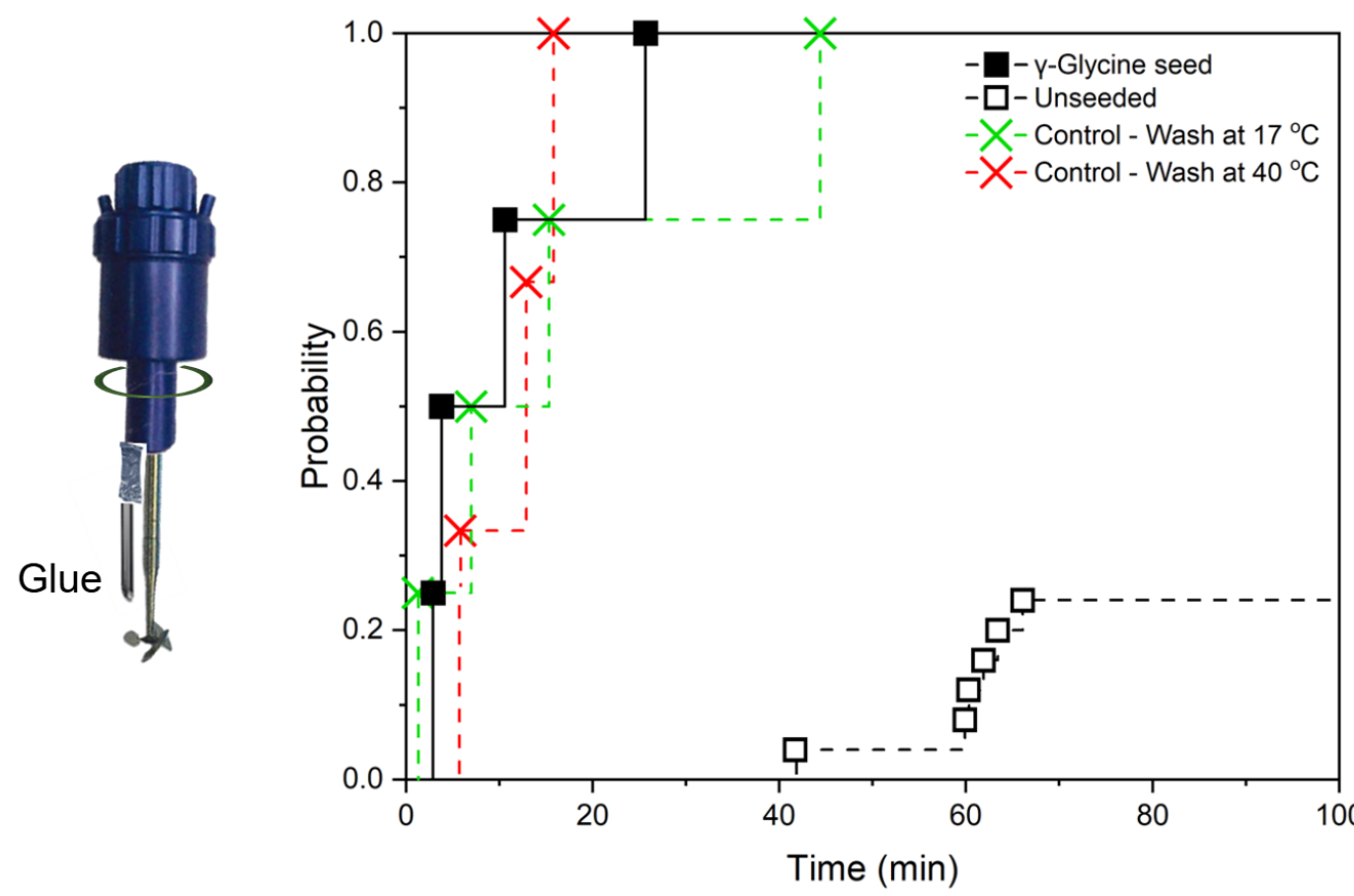

Figure 6 Probability distributions of induction times from control experiments conducted with a pre-dissolved solution and an O-ring at 20 °C, 320 mg/mL, and 700 rpm. Capillary tubes (with glue at the tip in all cases) were washed with deionized water at 17 °C and 40 °C. Green crosses represent control experiments where capillary tubes were washed with cold water; red crosses indicate experiments where capillary tubes were washed with warm water. Black filled squares correspond to delay times from seeded crystallizations with γ-glycine, while black open squares indicate induction times from unseeded crystallizations under the same conditions.

As shown in this figure, induction times from control experiments overlapped with delay times from seeded crystallizations even if internal encrustation was mitigated.This overlap was observed whether the capillary tubes were subjected to washing protocols with either cold or warm water. However, no standardized stirrer washing procedure was implemented yet, suggesting that the applied washing protocols were insufficient to fully eliminate residual contaminants on the stirrer or capillary tube surfaces. Additionally, differences in temperature may have contributed to the short induction times observed in the control experiments washed with cold water.

On the other hand, while the use of warm water in seeded crystallizations facilitates partial seed dissolution, which can be beneficial to avoid initial breeding, it may also lead to shorter induction times in control experiments by dissolving the glue that is used to secure the seed to the capillary tube. Nevertheless, the effect of dissolved glue still remains unknown. Overall, washing the overhead stirrers and capillary tubes with deionized water, before being introduced into the supersaturated solution, was not enough to avoid this overlap in delay and induction times. Therefore, additional control experiments with a standard stirrer washing procedure were performed.

S3.3 Effect of a Standardized Stirrer Washing Procedure

To assess the impact of cap and stirrer replacement at the seeding point on induction times in isolation from other materials in contact with the solution, control experiments in the absence of capillary tubes were conducted with and without a standardized stirrer washing procedure (SWP). The probability distributions of induction time from these controls are shown in Figure 7 below.

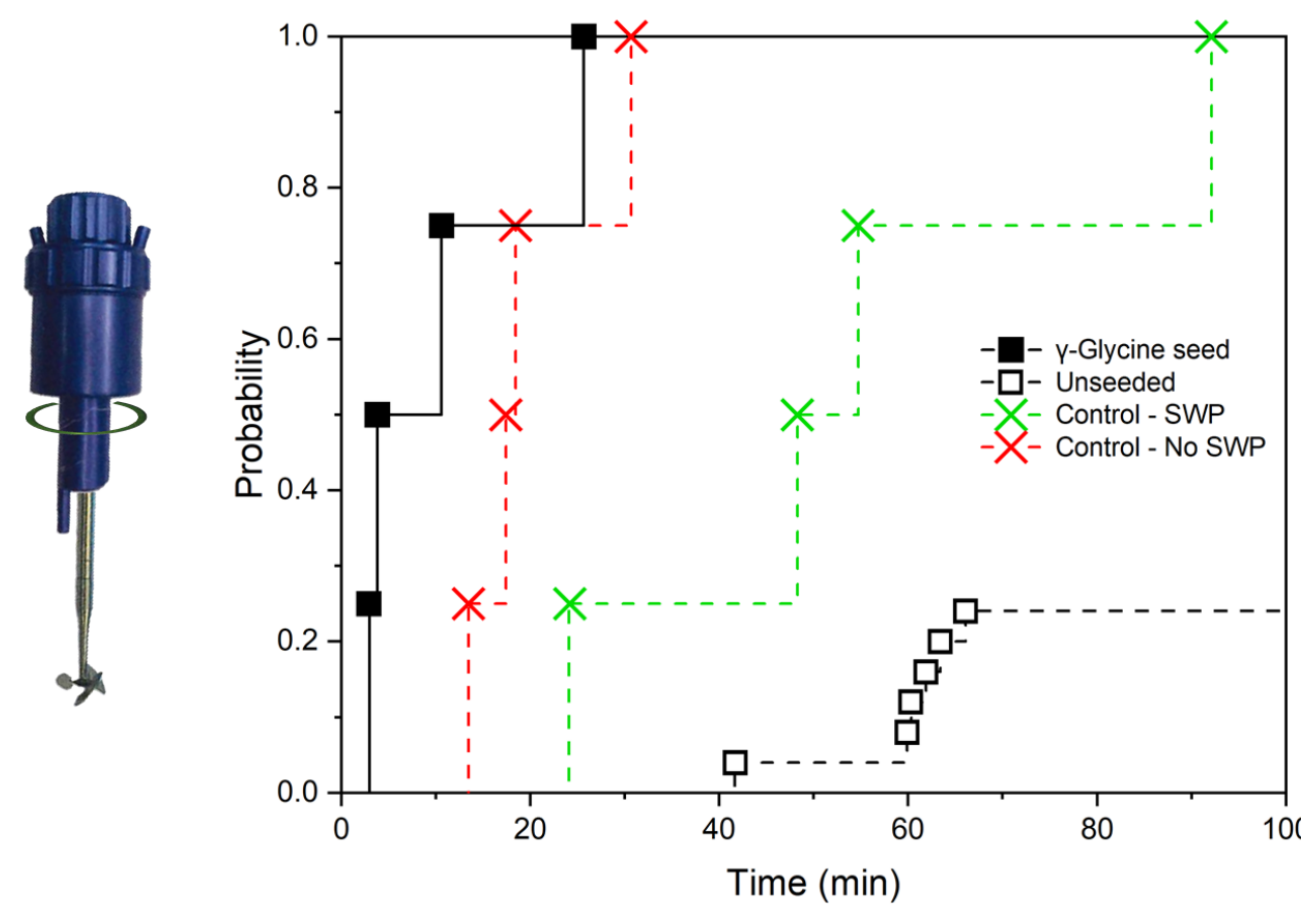

Figure 7 Probability distributions of induction times from control experiments without capillary tubes conducted with a pre-dissolved solution and an O-ring at 20 °C, 320 mg/mL, and 700 rpm. Green crosses represent control experiments where overhead stirrers were subjected to a stirrer washing procedure; red crosses indicate experiments where overhead stirrers were not subjected to a stirrer washing procedure. Black filled squares correspond to delay times from seeded crystallizations with γ-glycine, while black open squares indicate induction times from unseeded crystallizations without a change of caps under the same conditions.

In this figure, the effect of stirrer washing procedure can be clearly observed as longer induction times were obtained in control experiments that were subjected to an SWP (green crosses). Therefore, without proper stirrer washing that mimics the temperature profile of the solution, overhead stirrers may induce temperature fluctuations or introduce contaminants which can accelerate crystallization. For this reason, induction times from control experiments that were not subject to a stirrer washing procedure (red crosses) still overlapped with delay times from seeded crystallizations. Therefore, earlier nucleation due to the lack of an SWP could then be misinterpreted as secondary nucleation in seeded experiments where overhead stirrers have not been washed thoroughly.

S3.4 Effect of Parafilm in Contact with the Solution

After confirming the importance of a standardized stirrer washing procedure, the influence of other components in contact with the solution was also investigated. In control experiments, parafilm has been used so far to secure a capillary tube to the Crystalline cap, but the effect of this material in contact with the solution remains unknown. However, glycine has been previously reported to exhibit heterogeneous nucleation on hydrophobic surfaces such as PTFE[?] . Therefore, given that parafilm is also hydrophobic, its presence poses a risk of inducing unintended heterogeneous nucleation in seeded crystallizations where this material is also in contact with the solution.

To isolate the effect of parafilm, additional control experiments were conducted in the absence of capillary tubes but with parafilm positioned at the tip of the Crystalline cap to ensure direct exposure to the solution. The probability distribution of induction times from control experiments with this set-up (with and without an SWP) are presented in Figure 8 below.

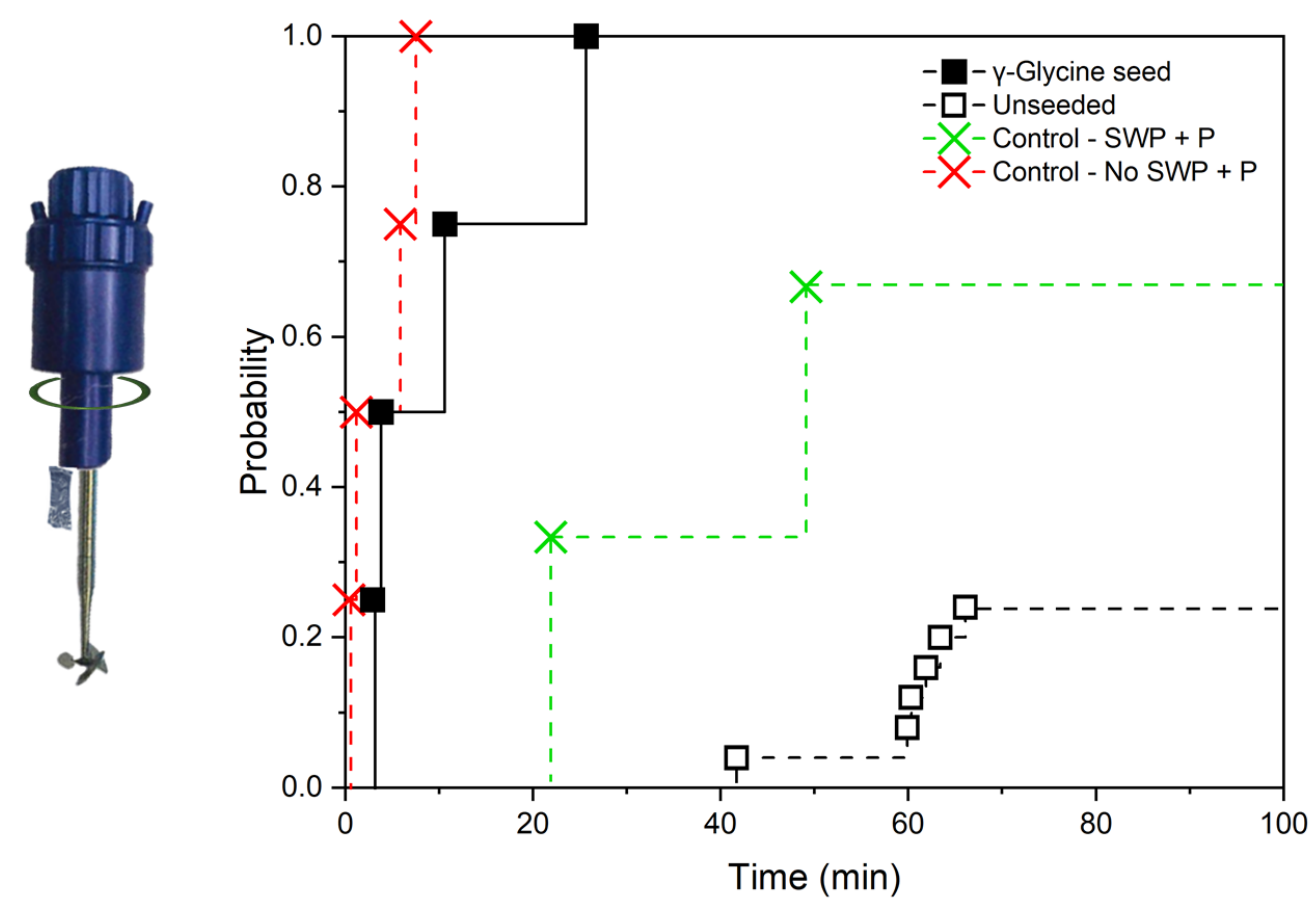

Figure 8 Probability distributions of induction times from control experiments using only parafilm, conducted with a pre-dissolved solution and an O-ring at 20 °C, 320 mg/mL, and 700 rpm. Green crosses represent control experiments where overhead stirrers were subjected to a stirrer washing procedure; red crosses indicate experiments where overhead stirrers were not subjected to a stirrer washing procedure. Black filled squares correspond to delay times from seeded crystallizations with γ-glycine, while black open squares indicate induction times from unseeded crystallizations without a change of caps under the same conditions.

As shown in this figure, the presence of parafilm in contact with the solution, in the absence of a standardized stirrer washing protocol, resulted in shorter induction times than even those observed in seeded crystallizations.This suggests that parafilm may facilitate heterogeneous nucleation in aqueous glycine solutions or that residual contaminants on the parafilm or stirrer could be responsible for earlier crystallization.

However, when a standardized stirrer washing procedure was conducted (green crosses in Figure 8) this earlier crystallization was also mitigated even in the presence of parafilm. Nevertheless, in seeded crystallizations, parafilm is used to secure the capillary tube containing the seed crystal at its tip. Therefore, subjecting the capillary tube secured with parafilm to an SWP would not be viable since an immersion of the seed in deionized water would lead to its complete dissolution.

For this reason, in the refined methodology reported previously, parafilm was not used to secure the capillary tube in place. Instead the seed-on-a-stick was located on the opposite side of the Crystalline cap through an alternative opening that did not require the use of this material to be in contact with the solution, as explained in the following section.

S3.5 Refined Methodology

In this last set of control experiments, overhead stirrers were subject to a standardized stirrer washing procedure and capillary tubes with glue at the tip were washed with deionized water at ambient temperature before being fitted in the Crystalline cap through an alternative opening. The probability distribution of induction times obtained from control experiments with this refined methodology (RF) is reported in Figure 9 below.

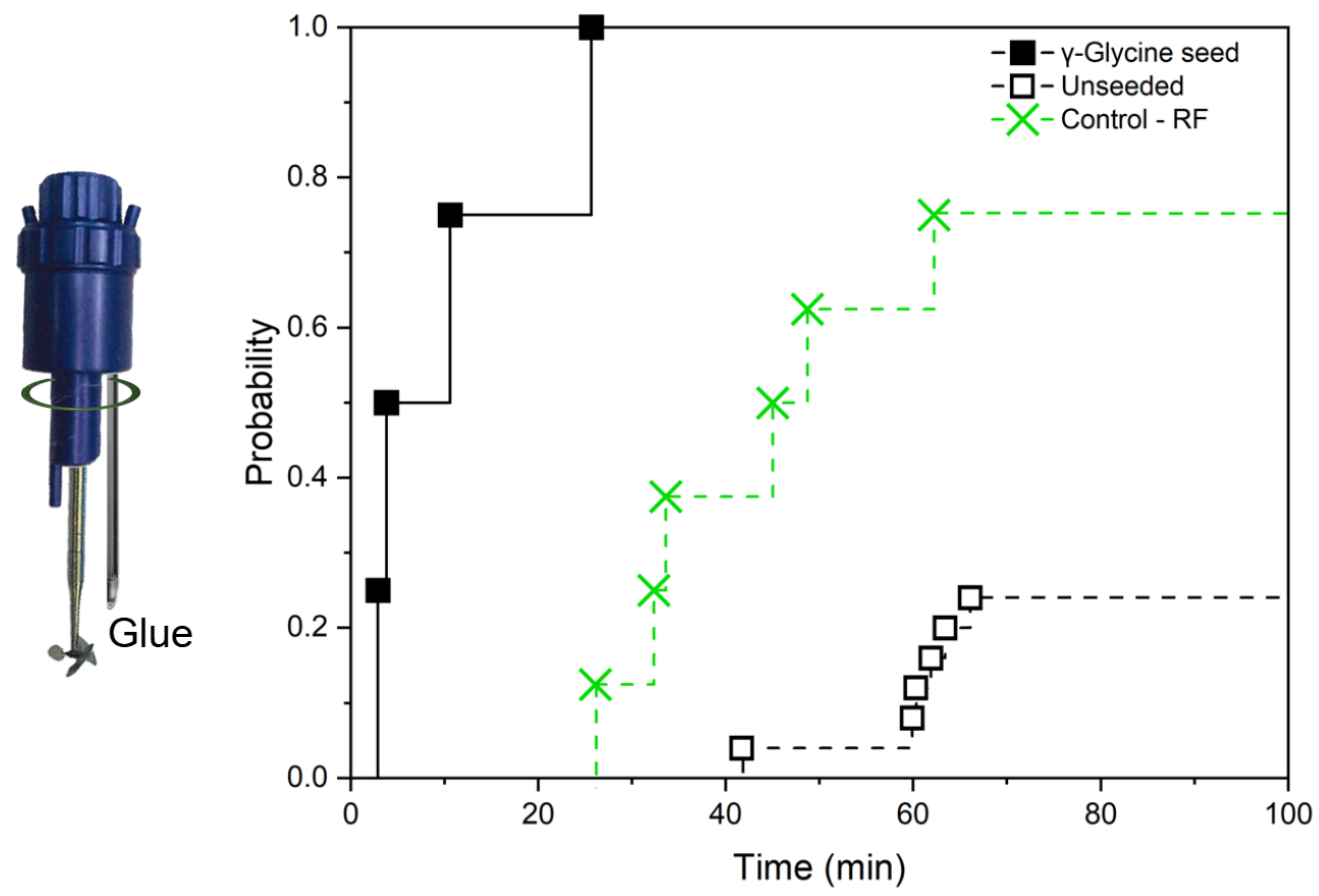

Figure 9 Probability distributions of induction times from control experiments using a refined methodology at 20 °C, 320 mg/mL, and 700 rpm. Controls were performed exclusively with glue at the tip of capillary tubes, and no parafilm was in contact with the solution. Green crosses represent control experiments where overhead stirrers were subjected to a stirrer washing procedure. Black filled squares correspond to delay times from seeded crystallizations with γ-glycine, while black open squares indicate induction times from unseeded crystallizations without a change of caps under the same conditions.

As shown in this figure, the refined control methodology effectively eliminated the overlap between induction times from control experiments and delay times from seeded crystallizations. Importantly, all seeded crystallizations performed previously were conducted with an analogous protocol with the only difference that a seed was also glued at the tip of the capillary tube.

This confirms that the modifications to the experimental protocol successfully mitigated nucleation artifacts that could have otherwise led to the misinterpretation of these effects as secondary nucleation in our seeded runs. Overall, the systematic refinement of the experimental protocol through successive control experiments ensures that the shorter delay times observed in seeded crystallizations with glycine in the main text are due to fluid-shear-driven secondary nucleation mechanisms alone rather than unintended nucleation from other sources.

S4 Delay and Induction Times Measured by Transmissivity

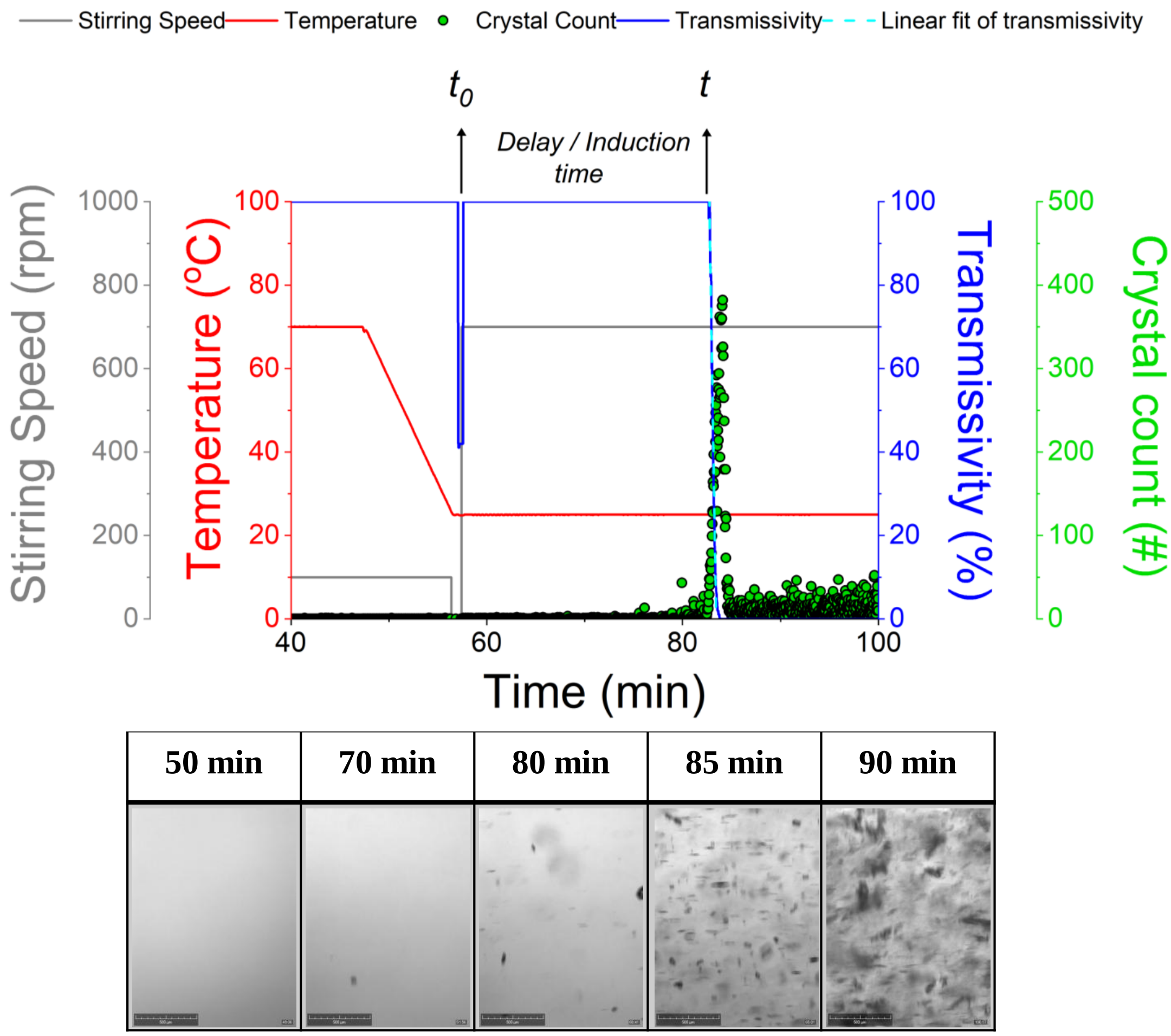

Figure 10 Transmissivity, temperature, stirring speed, and crystal count profiles obtained from the Crystalline® instrument. The onset of crystallization was determined by applying a linear fit to the region corresponding to the decrease in transmissivity and calculating its intersection with a transmissivity value of 100%, representing a fully dissolved solution.

S5 Estimation of Secondary Nucleation Rates

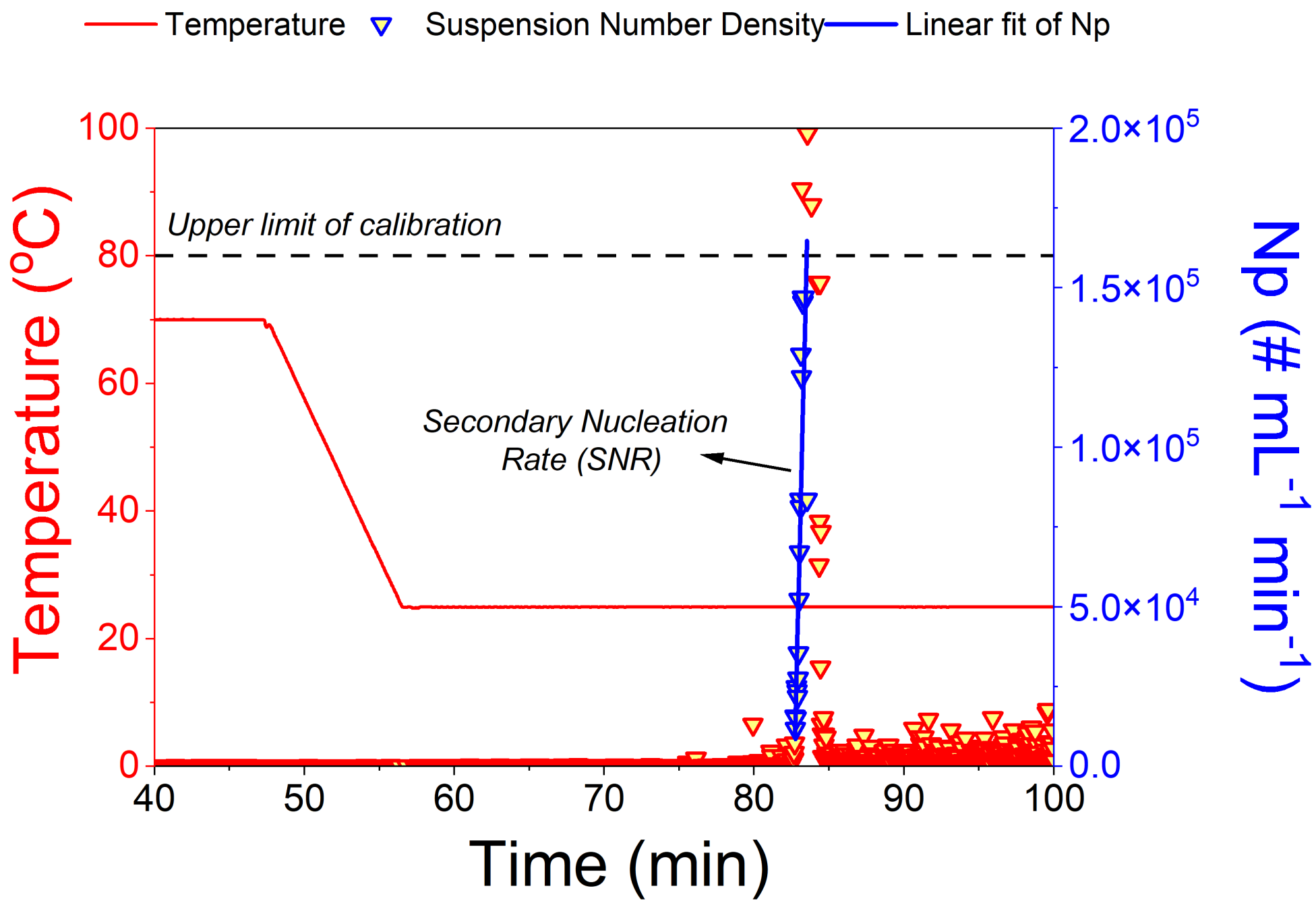

Figure 11 Temporal evolution of the suspension number density superimposed on the temperature profile. The suspension number density was estimated directly from crystal counts obtained using the Crystalline® instrument, based on a previously established calibration with polystyrene spheres. Secondary nucleation rates were determined from the slope of the linear fit to the increasing region of the suspension number density. All analyses were conducted within the validated range of the polystyrene sphere calibration to avoid extrapolation beyond its upper limit.

S6 Suspension Number Density Profiles for Seed-on-a-Stick, Control, and Unseeded Experiments

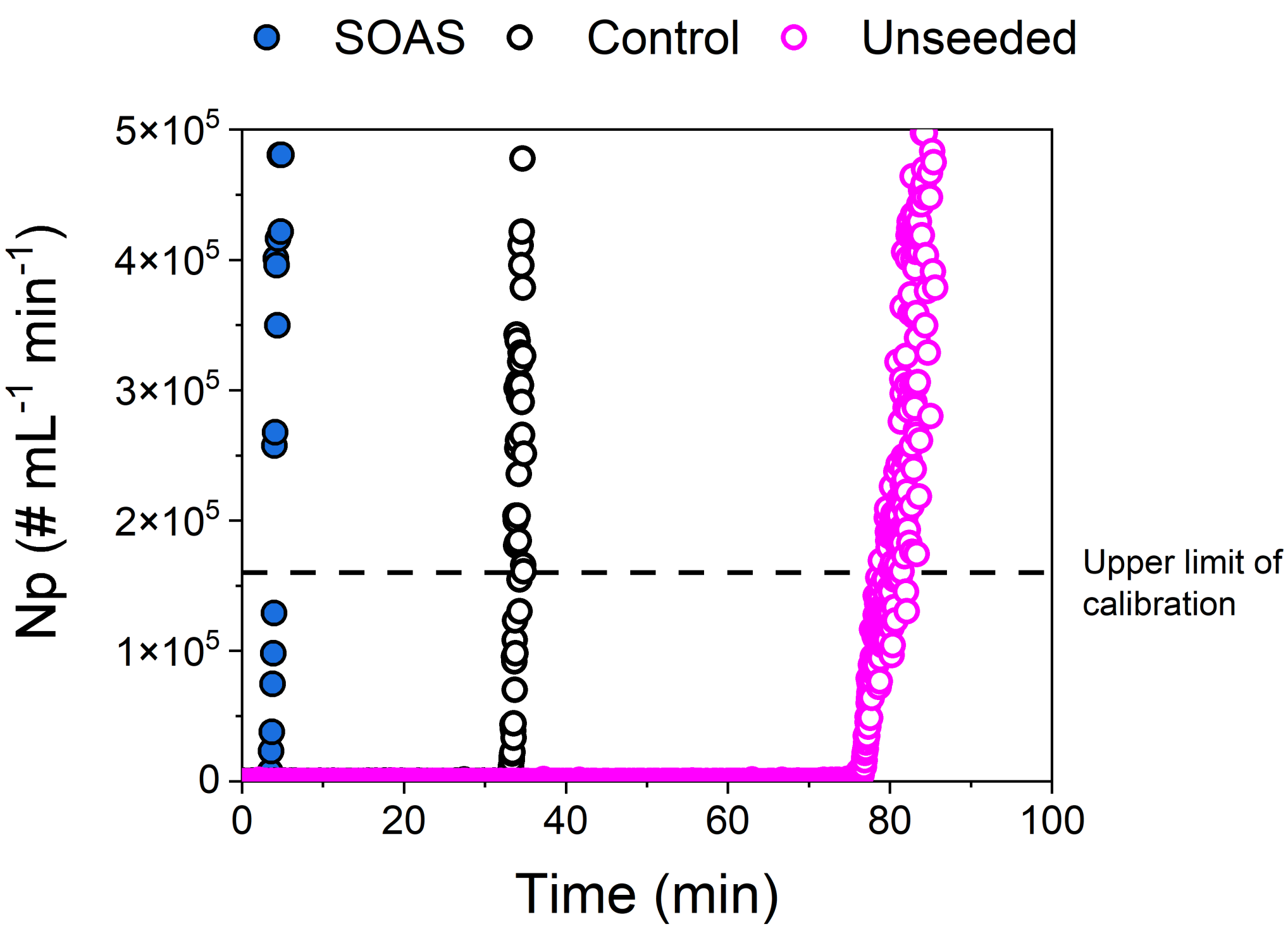

Figure 12 Comparison of the temporal evolution of the suspension number density across seed-on-a-stick, control, and unseeded experiments conducted at 320 g g^{-1} and 20 °C. A time of zero corresponds to the moment when isothermal conditions were established, enabling direct comparison between the different experimental configurations.
